# Automated Insulin Delivery with Remote Real-Time Continuous Glucose Monitoring for Hospitalized Patients with Diabetes: A Multicenter, Single-Arm, Feasibility Trial

**DOI:** 10.1089/dia.2023.0304

**Published:** 2023-10-12

**Authors:** Georgia M. Davis, Michael S. Hughes, Sue A. Brown, Judy Sibayan, M. Citlalli Perez-Guzman, Meaghan Stumpf, Zachary Thompson, Marina Basina, Ronak M. Patel, Joi Hester, Amalia Abraham, Trang T. Ly, Cherie Chaney, Marilyn Tan, Liana Hsu, Craig Kollman, Roy W. Beck, Rayhan Lal, Bruce Buckingham, Francisco J. Pasquel

**Affiliations:** ^1^ Division of Endocrinology, Metabolism, and Lipids, Department of Medicine, Emory University School of Medicine, Atlanta, Georgia, USA.; ^2^ Division of Endocrinology, Gerontology and Metabolism, Department of Medicine, Stanford University, Stanford, California, USA.; ^3^ Division of Endocrinology, Center for Diabetes Technology, University of Virginia, Charlottesville, Virginia, USA.; ^4^ Jaeb Center for Health Research, Tampa, Florida, USA.; ^5^ Insulet Corporation, Acton, Massachusetts, USA.; ^6^ Division of Pediatric Endocrinology, Department of Pediatrics, Stanford University, Stanford, California, USA.

**Keywords:** Automated insulin delivery, Hybrid closed-loop, Inpatient diabetes

## Abstract

**Introduction:**

Multiple daily injection insulin therapy frequently fails to meet hospital glycemic goals and is prone to hypoglycemia. Automated insulin delivery (AID) with remote glucose monitoring offers a solution to these shortcomings.

**Research Design and Methods:**

In a single-arm multicenter pilot trial, we tested the feasibility, safety, and effectiveness of the Omnipod 5 AID System with real-time continuous glucose monitoring (CGM) for up to 10 days in hospitalized patients with insulin-requiring diabetes on nonintensive care unit medical–surgical units. Primary endpoints included the proportion of time in automated mode and percent time-in-range (TIR 70–180 mg/dL) among participants with >48 h of CGM data. Safety endpoints included incidence of severe hypoglycemia and diabetes-related ketoacidosis (DKA). Additional glycemic endpoints, CGM accuracy, and patient satisfaction were also explored.

**Results:**

Twenty-two participants were enrolled; 18 used the system for a total of 96 days (mean 5.3 ± 3.1 days per patient), and 16 had sufficient CGM data required for analysis. Median percent time in automated mode was 95% (interquartile range 92%–98%) for the 18 system users, and the 16 participants with >48 h of CGM data achieved an overall TIR of 68% ± 16%, with 0.17% ± 0.3% time <70 mg/dL and 0.06% ± 0.2% time <54 mg/dL. Sensor mean glucose was 167 ± 21 mg/dL. There were no DKA or severe hypoglycemic events. All participants reported satisfaction with the system at study end.

**Conclusions:**

The use of AID with a disposable tubeless patch-pump along with remote real-time CGM is feasible in the hospital setting. These results warrant further investigation in randomized trials.

## Introduction

Glycemic control can be difficult to achieve in the hospital with current management strategies, leading to increased rates of hospital complications, prolonged hospitalizations, and mortality.^1,2^ Current standards of care for management of inpatient diabetes and hyperglycemia continue to rely on subcutaneous insulin regimens, most commonly with a combination of basal and rapid-acting insulin analogues.^3,4^ Despite frequent monitoring with finger-stick capillary blood glucose (CBG) testing and insulin dose adjustments, multiple daily injection (MDI) insulin regimens often fail to achieve desired glycemic control and lead to iatrogenic hypoglycemia.^1,5^ Furthermore, both health care staff and patients are burdened by the frequent monitoring and management required by these regimens.^6,7^

Outpatient use of diabetes technologies, particularly continuous glucose monitoring (CGM) and automated insulin delivery (AID) systems, rapidly expanded and has generated significant interest in its translation into the hospital setting. During the COVID-19 pandemic, accelerated adaptation of outpatient diabetes technology for inpatient use occurred in multiple health systems.^8,9^ Hybrid protocols, combining CGM with periodic CBG testing, were implemented by us and others to use CGM reliably in the hospital for patients under isolation precautions.^10–12^ Although adding CGM may reduce nursing staff burdens and reduce the risk for hypoglycemia in select high-risk populations,^13,14^ the impact of CGM on achieving glycemic targets when compared with traditional CBG testing alone appears to be very modest.^13–16^ Therefore, there is a need to develop technology-based approaches that further optimize glycemic control while also catering to the specific hospital implementation needs, including active nursing-staff involvement and the use of devices with remote monitoring capabilities.

The promise of using closed-loop or AID systems in the hospital lies in their ability to react in real time to CGM values and automatically adjust insulin infusion based on internal predictive algorithms to maintain a target range of glycemic control. Small, high-quality, European studies using the CamAPS HX algorithm with a tubed insulin pump in the hospital have shown consistent improvements in time-in-range (TIR) compared with standard-of-care MDI insulin regimens.^17–19^ These data suggest that using AID can improve glycemic control and avoid iatrogenic hypoglycemia in noncritically ill hospitalized patients. Such findings, however, have not yet been reproduced with other AID systems.

In addition, there are unknowns regarding implementation strategies, including end-user involvement (i.e., nursing staff, hospital care teams), costs, adoption, and scalability in the hospital, although some recent work has been done to this end.^20^ Furthermore, there are concerns about the reliability of CGM in the inpatient setting due to mechanical, hemodynamic, and biochemical interferences.^10,21,22^

Building upon experience gained with remote glucose monitoring before and during COVID-19, we designed a feasibility study to test an AID system in the hospital. We selected the preclearance Omnipod 5 AID system, which paired with the Dexcom G6 sensor, allowing for integrated single-use of disposable devices with remote monitoring capabilities. We implemented a nurse-driven protocol for remote insulin delivery, glucose telemetry with alarms, a protocol for CGM accuracy validation, and electronic health record (EHR) documentation at three different health care systems. In this study, we present the results of our single-arm multicenter feasibility trial aimed at determining the functional operability of an AID system and its effect on glycemic control in a diverse insulin-requiring inpatient population.

## Research Design and Methods

### Study design and participants

AIDING (Automated Insulin Delivery for INpatients with dysGlycemia) feasibility was a single-arm, multicenter, stepwise, open-label trial at three U.S. academic medical centers: Emory University, Stanford University, and the University of Virginia. Eligible participants were ≥18 years of age with type 1 diabetes (T1D) or type 2 diabetes (T2D) requiring inpatient insulin therapy and admitted to a general medical–surgical hospital service. Key exclusion criteria were as follows: anticipated length of hospital stay <48 h, insulin total daily dose (TDD) >100 units, current use of hydroxyurea or >4 g/day acetaminophen, COVID-19 infection, evidence of hemodynamic instability or hyperglycemic crises (diabetes-related ketoacidosis [DKA] or hyperosmolar hyperglycemic state), hypoxia (oxygen saturation <95% on supplemental oxygen), clinically significant liver failure, estimated glomerular filtration rate <30 mL/min/1.73 m^2^, hemoglobin <7 g/dL, currently pregnant or breastfeeding, unable or unwilling to use rapid-acting insulin analogues, and presence of a condition impeding ability to consent or answer questionnaires.

Approval was obtained from the U.S. Food and Drug Administration (IDE G200280), the U.S. Centers for Medicare and Medicaid Services, and each institution's local Institutional Review Board (IRB) as well as a central IRB through the data coordinating center, the Jaeb Center for Health Research (JCHR). Study candidates were identified by a daily chart review and provided written informed consent for enrollment.

### Procedures

#### System setup and use

All enrolled participants used the preclearance Omnipod 5 AID system (Insulet Corporation, Acton, MA), which utilizes a Dexcom G6 CGM (Dexcom, Inc., San Diego, CA) for inpatient glucose management until discharge or for a maximum of up to 10 days. The system was managed by the study investigators and trained nurses. Throughout the study, participants did not interact with the system interface. The Controller, a locked-down Samsung smartphone used as the device interface and receiver, was kept in a locked box in the room and used by nurses for meal boluses. The Pod and sensor are attached to the patient with the algorithm on the Pod, so the Controller is not necessary for AID; it is only needed to deliver a bolus or start/stop a Pod pump. The devices were set up, initiated, placed, and exchanged as needed by study investigators. Initial target glucose was set at 120 mg/dL (6.7 mmol/L).

Other insulin pump settings were not expressly protocolized; however, investigators typically aimed to use either 60% of current insulin TDD or 50% of weight-based TDD to determine initial basal rate, unless clinically inappropriate (e.g., patient's current insulin dose needed adjustment). All settings were reviewed at least once every 24 h, and any adjustments were made by investigators.

**FIG. 1.**
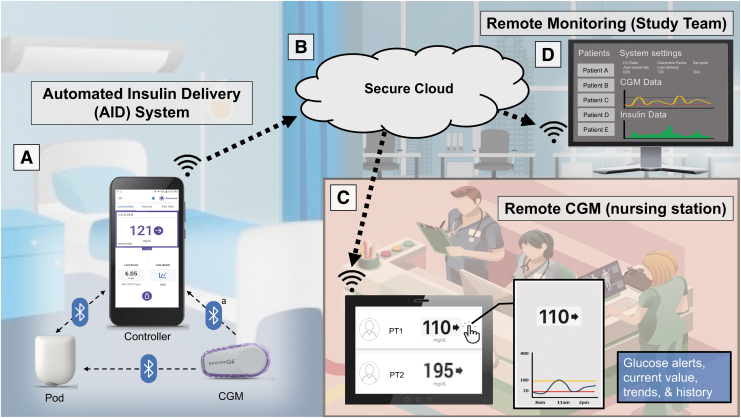
Automated insulin delivery system set up in the hospital for the AIDING study. **(A)** The devices all communicated via Bluetooth in the patient room, primarily managed by the bedside nurse. However, we did test the ability to manage interaction with the system using the phone-based Controller from outside the rooms. **(B)** The data wirelessly streams to a secure cloud via cell phone signal. **(C)** The cloud connected wirelessly to the Dexcom Follow application on an iPad at the nursing station, which provided real-time glucose telemetry at the nursing unit. This was programmed to alarm for any glucose drop below 80 mg/dL (4.4 mmol/L) and for prolonged glucose level above 300 mg/dL (16.7 mmol/L) for more than 2 h. CGM values below 80 mg/dL were assessed using finger-stick CBG and hypoglycemia was treated according to each institutional protocol, which was indicated for glucoses <70 mg/dL (3.9 mmol/L). Guidance for interventions to prevent hypoglycemia (e.g., small amount of juice for glucose just above lower limit but accompanied by a downward trend arrow) was neither provided by the protocol nor was it prohibited. Prolonged hyperglycemia necessitated reaching out to study for advice on correction bolusing, system troubleshooting, or setting adjustments, as well as increased frequency of CBG assessments until glucose dropped back below 300 mg/dL. **(D).** Data were also available continuously to the study via the cloud in the Dexcom Follow application, the Dexcom Clarity application, and Insulet's investigational data management software. a. The figure depicts CGM connection to the Controller because this was used on the study devices; however, it should be noted that the commercial release of the product does not connect the Controller (instead it connects to the patient's personal cell phone or CGM receiver). AIDING, Automated Insulin Delivery for INpatients with DysGlycemia; CBG, capillary blood glucose; CGM, continuous glucose monitoring.

Glucose data were monitored remotely through the Dexcom Follow app on a dedicated tablet at the unit nursing station and by study personnel (Fig. 1). CGM alerts were programmed for CGM values <80 mg/dL (4.4 mmol/L). Response to this alarm included assessment of concurrent CBG and treatment according to institutional hypoglycemia protocol if the CBG test confirmed glucose was <80 mg/dL. Investigators received additional alerts for CGM glucose values >250 mg/dL (13.9 mmol/L) lasting >1 h and advised the nurse to give corrective insulin dosing if appropriate. Unexplained hyperglycemia with CGM values >300 mg/dL (16.7 mmol/L) for >60 min required a correction insulin dose and triggered increased frequency of finger-stick assessments (hourly for T1D or every 2 h for T2D) until CGM <300 mg/dL. Serum beta hydroxybutyrate was analyzed if clinical symptoms concerning ketoacidosis were present.

Continuous real-time insulin pump data were available to study personnel through the manufacturer's investigational data management software.

#### Nurse training

Each site worked with nursing managers and educators to facilitate study awareness and general understanding of the protocol and objectives. Every nurse caring for an enrolled study patient for the first time received “just-in-time” training^23^ at shift start. Just-in-time trainings lasted 10–20 min and focused on nursing responsibilities, including basics of AID and Omnipod 5 system function, how to perform CGM validations, when to remove/discontinue the system, how to respond to hyper- and hypoglycemia alarms, and how to perform actions such as bolusing, verifying insulin delivery, assessing system mode, and reviewing settings. Insulin boluses were given remotely by the nurses using the Controller. Nurses were only required to sign off on training once, but refresher training was offered and always available for any nurse as needed over the course of the study.

#### CGM accuracy surveillance and maintenance

Because the system uses CGM values to calculate insulin delivery, CGM accuracy was assessed with bedside CBG values in an ongoing “validation” process (the glucometers used included the Nova StatStrip; Emory/Grady and UVA) and Abbott Freestyle Precision Pro (Stanford). A CGM was considered successfully validated if it was within ±20% of the simultaneous reference CBG for values ≥70 mg/dL (3.9 mmol/L) or within ±20 mg/dL (1.1 mmol/L) for values <70 mg/dL. Following the FDA's recommendations, the study was divided into two phases, with phase 1 (*n* = 9) requiring six CGM validations per day and phase 2 (*n* = 9) requiring four CGM validations per day.

**FIG. 2.**
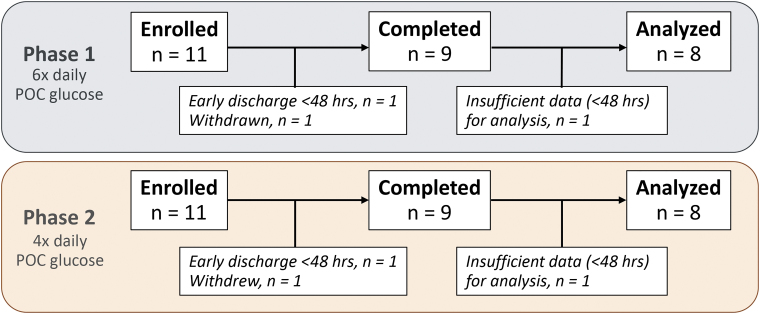
Study flow diagram. Enrollment was performed in two phases. In phase 1, finger-stick CBG was assessed and compared with simultaneous CGM value (using validation process described in the Research Design and Methods section) six times daily, before breakfast, before lunch, before dinner, before bedtime, and two times overnight. After data safety monitoring board review, the study moved into phase 2, which reduced the daily number of CBG checks and validations to four by removing the two overnight assessments. In each phase, 11 patients were enrolled, 1 patient was discharged within 48 h of enrollment, and 1 patient was withdrawn from the study. In phase 1, one participant was withdrawn due to initiation of high-dose (≥4 g daily) acetaminophen. Nine participants from each phase completed the study, one of whom from each phase did not have the prespecified 48 h of CGM data required for inclusion in glycemia analysis. Therefore, 16 participants in total were included in the final glycemia analysis.

Transition from phase 1 to phase 2 occurred after data safety monitoring review (Fig. 2). Bedside nurses performed CGM validations in a dedicated EHR flow sheet, which automatically assessed validation criteria, with measurements before meals and at bedtime, and an additional two checks overnight during phase 1. Nurses were advised to avoid validating during periods of rapid glucose change >2 mg/dL (0.1 mmol/L) per minute.

Successful validation was required for any new CGM sensor before automated mode could be initiated. If any subsequent validation was unsuccessful, the system could remain in automated mode or could be switched to manual mode at the investigator's discretion. To administer CGM-based insulin boluses, a successful validation from the most recent scheduled time was required. Unsuccessful validations triggered CBG testing for any insulin bolus calculation until CGM validation was successful again. Calibrations were avoided, if possible, in the first 12 h after CGM warm-up. Sensor exchange was considered if two sequential validations failed after a calibration and required if 24 h elapsed without a successful validation.

#### Study completion and system discontinuation

Enrolled participants were started on the Omnipod 5 AID system and continued use for up to 10 days during hospitalization or until hospital discharge, whichever occurred first. Early study termination was defined by wear of the AID system for less than the prespecified time frame of 48 h. The entire system was removed for magnetic resonance imaging, but only the pod (not the CGM) required removal for computed tomography. Transfer to the intensive care unit (ICU), high-dose acetaminophen (>4 g/day) use, hydroxyurea use, or lack of CGM validation for 48 h after enrollment required system discontinuation and termination of study participation.

### Data collection and endpoints

Ongoing data collection included all administered medications, hematocrit, creatinine, CGM data (via Dexcom Clarity), and Omnipod 5 insulin delivery data (via Insulet's investigational data management software). At study end, participants completed a brief questionnaire assessing perceptions of inpatient use of the device, including open-ended questions inquiring about likes, dislikes, and suggestions for improvement (Supplementary S1).

### Statistical analysis

As a pilot and feasibility trial, this study was not intended to draw statistical conclusions and did not present a formal statistical hypothesis due to its exploratory nature. The sample size for this study was based upon a convenience sample of up to 30 participants, with the aim of enrolling at least 18 participants to receive AID therapy for at least 48 h.

#### Outcome measures

Primary endpoints were (1) the proportion of time spent in automated mode (functional operability) and (2) the percent TIR (glycemic control), defined as 70–180 mg/dL (3.9–10 mmol/L). Both endpoints were calculated from the time of initial CGM validation until the following: ICU admission, hospital discharge, or 10 days following initial CGM validation. For inclusion in CGM glycemic analyses, a minimum of 48 h of CGM data was required.

Secondary and exploratory endpoints related to system function, additional glycemic metrics, and patient perceptions were also assessed. System function metrics included time from enrollment to automated mode initiation, use of system modes and settings, and CGM accuracy. Additional glycemic metrics included the percentages of time spent <54 mg/dL (<3 mmol/L), 54–69 mg/dL (3–3.9 mmol/L), 181–250 mg/dL (10.1–13.9 mmol/L), and >250 mg/dL (>13.9 mmol/L).^24^ Patient perceptions of AID use were assessed with a combination of Likert scale and open-ended questions.

This trial is registered with ClinicalTrials.gov, NCT04714216.

## Results

Between June 29, 2021, and August 8, 2022, 22 participants consented for participation. One participant was not started on AID due to initiation of high-dose acetaminophen, and one participant was withdrawn due to clinical conditions impacting CGM reliability. Two patients were discharged before completing 48 h. Eighteen participants stayed for ≥48 h on the AID device and completed the study; however, two were excluded from glycemic analyses because they lacked the prespecified requirement of ≥48 h of active CGM data (Fig. 2).

Of the 18 participants, most were male and ranged 32–83 years in age. Half identified as non-Hispanic white, and 39% identified as black/African American. About two-thirds reported a yearly household income of less than $100,000, with 28% reporting less than $20,000. Most participants were on Medicaid or other government-sponsored insurance. Two participants (11%) had T1D and the remaining 16 (89%) had T2D; most had diabetes for at least 10 years. Mean hemoglobin A1c (HbA1c) was 8.2% ± 1.4%. Half had never used a CGM before, and 83% were on home insulin therapy via injection(s). All participants were naive to insulin pump therapy. Average insulin TDD before enrollment was 0.5 ± 0.2 units/kg/day. The length of hospitalization varied widely, ranging from 4 to 50 days, with a median of 12 days. Full participant characteristics are detailed in Table 1.

**Table 1.** Participant Characteristics

	Phase 1 (*n* = 9)	Phase 2 (*n* = 9)	Overall (*N* = 18)
Age, years			
Mean ± SD	63 ± 13	55 ± 15	59 ± 14
Range	34–83	32–81	32–83
Female sex, *N* (%)	2 (22)	3 (33)	5 (28)
Racial and ethnic background, *N* (%)			
Black/African American	4 (44)	3 (33)	7 (39)
Hispanic or Latino/a	0	1 (11)	1 (6)
White, non-Hispanic	4 (44)	5 (56)	9 (50)
More than one^a^	1 (11)	0	1 (6)
Body mass index, kg/m^2^			
Median (IQR)	30.0 (27.4, 33.0)	33.3 (26.6, 35.9)	31.2 (26.6, 35.0)
Range	25.7–36.5	18.6–50.4	18.6–50.4
Highest level of education, *N* (%)			
Less than high school diploma	4 (44)	4 (44)	8 (44)
High school completion or equivalent	0	0	0
Associate degree/some college	4 (44)	2 (22)	6 (33)
Bachelor's degree	1 (11)	3 (33)	4 (22)
Annual household income (USD), *N* (%)			
< $20,000	1 (11)	4 (44)	5 (28)
$20,000–$49,999	2 (22)	1 (11)	3 (17)
$50,000–$99,999	2 (22)	2 (22)	4 (22)
≥ $100,000	1 (11)	1 (11)	2 (11)
Does not wish to report	3 (33)	1 (11)	4 (22)
Health insurance type, *N* (%)			
Private	3 (33)	3 (33)	6 (33)
Medicaid or other government-sponsored	5 (56)	6 (67)	11 (61)
Military	1 (11)	0	1 (6)
Type of diabetes, *N* (%)			
Type 1 diabetes	0	2 (22)	2 (11)
Type 2 diabetes	9 (100)	7 (78)	16 (89)
Duration of diabetes, years			
Median (IQR)	15 (10, 20)	18 (15, 20)	18 (10, 20)
Range	2–62	0–26	0–62
Most recent HbA1c (within 1 month)			
Mean ± SD, % (mmol/mol)	7.8 ± 1.5 (62 ± 16)	8.6 ± 1.3 (70 ± 15)	8.2 ± 1.4 (66 ± 15)
Range, % (mmol/mol)	6.3–11 (45–97)	7.0–11 (53–97)	6.3–11 (45–97)
≥8.0% (≥64 mmol/mol), *N* (%)	4 (44)	5 (56)	9 (50)
Estimated frequency of SMBG, times/day			
Median (IQR)	3 (0, 4)	3.5 (2.5, 4)	3 (1, 4)
Range	0–4	0–5	0–5
History of continuous glucose monitor use			
Never	5 (56)	4 (44)	9 (50)
In past, but not current	1 (11)	4 (44)	5 (28)
Current (immediately before hospitalization)	3 (33)	1 (11)	4 (22)
Modality of home insulin delivery, *N* (%)			
Injection	6 (67)	9 (100)	15 (83)
Insulin pump	0	0	0
None of the above (not on insulin)	3 (33)	0	3 (17)
Insulin total daily dose, units/kg/day			
Mean ± SD	0.5 ± 0.2	0.5 ± 0.2	0.5 ± 0.2
Range	0.2–0.7	0.3–1.0	0.2–1.0
DKA in past 12 months^b^	1 (11)	0	1 (6)
Duration of hospital stay, days			
Median (IQR)	17 (11, 23)	11 (7, 26)	12 (9, 25)
Range	4–45	4–50	4–50
Primary reason for admission^c^			
Cardiac	4 (44)	2 (22)	6 (33)
Respiratory	1 (11)	1 (11)	2 (11)
Infectious	2 (22)	4 (44)	6 (33)
Glycemic	0	1 (11)	1 (6)
Other	2 (22)	1 (11)	3 (17)
On systemic steroids	4 (44)	3 (33)	7 (39)
Supraphysiologic doses^d^	3 (33)	0	3 (19)
CKD, baseline GFR <60 mL/min/1.73m^2^	6 (67)	4 (44)	10 (56)

^a^ One participant identified as Hispanic Asian.

^b^ One episode of DKA over the prior 12 months in one participant.

^c^ Admission diagnoses fall into several categories, namely: cardiac causes, which encompass stable angina (two cases), acute coronary syndrome, heart failure (two cases), and atrial fibrillation; respiratory causes, which comprise respiratory failure necessitating lung transplantation (two cases); infectious causes, which include pneumonia, infected groin hematoma, urinary tract infection, osteomyelitis, and diabetic foot infection (two cases); one glycemic cause, which was severe hypoglycemia; and other causes, which consist of postendoscopic retrograde cholangiopancreatography transaminitis, closed femoral fracture, and thrombophlebitis.

^d^ Defined as a total daily dose of >5 mg of prednisone, or alternative steroid equivalent. During the study period, one patient received prednisone 40 mg daily, one patient received prednisone 10 mg daily, and one participant received 500 mg daily methylprednisolone for 3 days.

DKA, diabetes-related ketoacidosis; GFR, glomerular filtration rate; HbA1c, hemoglobin A1c; IQR, interquartile range; SD, standard deviation; SMBG, self-monitoring of blood glucose; USD, U.S. dollars.

**FIG. 3.**
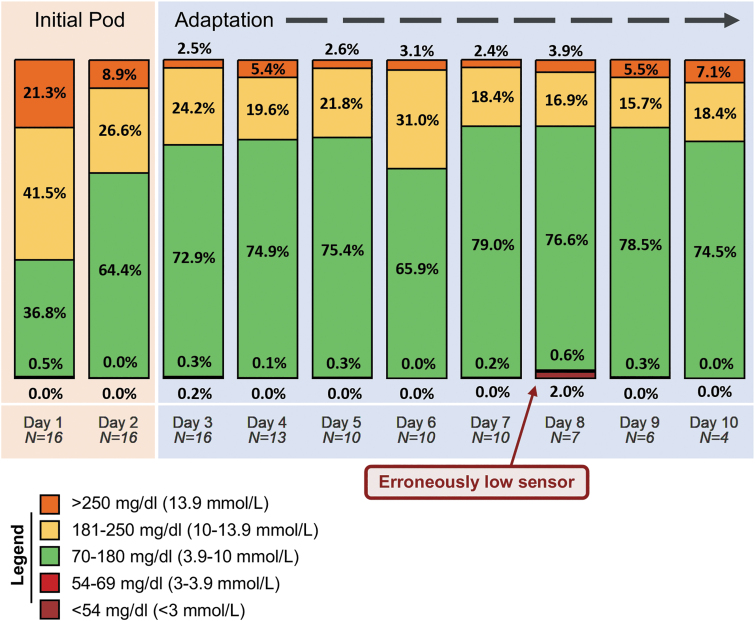
Average participant-aggregated glycemic control by day of study enrollment. Values are expressed as the average of the proportions of time each participant spent in each range on the specified day of enrollment. Each “day” represents a full 24-h period, and day 1 starts from the time of completion of initial continuous glucose monitor warm-up (i.e., days are not midnight-midnight). Day 8 includes data from one participant who had a sensor that was erroneously low, ranging 75–175 mg/dL below finger-stick CBG level on two consecutive scheduled checks. “Initial pod” and “adaptation” are behind the bars and highlight a specific action of the Omnipod 5 system initial start-up, in which the algorithmic basal dosing is approximately half as aggressive with the initial Pod. The algorithm subsequently increases its automated basal calculations with the first Pod change that occurs after 48 h on the system. Correspondingly, the bars in the figure show a notable improvement in glycemic control after this period.

For the full cohort of 18 participants, cumulative AID system use was 96 days (mean 5.3 ± 3.1 days per participant). Results for the primary endpoints are shown in Table 2, with median percent time in automated mode of 95% (interquartile range [IQR] 92%, 98%). For the *n* = 16 using CGM for ≥48 hrs, mean TIR was 68% ± 16%. Mean percent times <54 mg/dL (<3 mmol/L), 54–69 mg/dL (3–3.9 mmol/L), 181–250 mg/dL (10.1–13.9 mmol/L), and >250 mg/dL (>13.9 mmol/L) were 0.06% ± 0.2%, 0.17% ± 0.3%, 25% ± 11%, and 6.9% ± 7.0%, respectively; mean glucose was 167 ± 21 mg/dL (9.3 ± 1.2 mmol/L). Figure 3 displays the average time the cohort spent in each glycemic range by number of days on the system. Patient-level data are available in Supplementary S2.

**Table 2.** Feasibility and Efficacy Endpoints

Primary endpoints	
Percent time in automated mode	
Mean ± SD	91% ± 11%
Median (IQR)	95% (92%, 98%)
Range	60%–99%
Percent TIR, 70–180 mg/dL (3.9–10 mmol/L)^a,b^	
Mean ± SD	68% ± 16%
Median (IQR)	63% (58%, 80%)
Range	44%–95%

Secondary glycemic endpoints*^a,b^*	
Percent time in nontarget glycemic ranges	
>250 mg/dL (>13.9 mmol/L)	6.9% ± 7.0%
181–250 mg/dL (10.1–13.9 mmol/L)	25% ± 11%
54–69 mg/dL (3–3.9 mmol/L)	0.17% ± 0.3%
<54 mg/dL (<3 mmol/L)	0.06% ± 0.2%
CGM mean glucose, mg/dL (mmol/L)	167 ± 21 (9.3 ± 1.2)
Coefficient of variation	25% ± 5.5%
No. of episodes <70 mg/dL (<3.9 mmol/L) per patient	
Median (IQR)	0 (0, 0)
Range	0–2
No. of episodes <54 mg/dL (<3 mmol/L) per patient	
Median (IQR)	0 (0, 0)
Range	0–2

^a^ Includes only participants who met prespecified requiring ≥48 h of active CGM data for inclusion in glycemic analyses, *n* = 16. Mean ± SD TIR for the full 18 participants was 69% ± 15% (range 44%–95%).

^b^ CGM data are calculated from the first successful CGM accuracy validation, up to a maximum of 10 days.

CGM, continuous glucose monitoring; TIR, time-in-target glucose range 70–180 mg/dL (3.9–10 mmol/L).

**FIG. 4.**
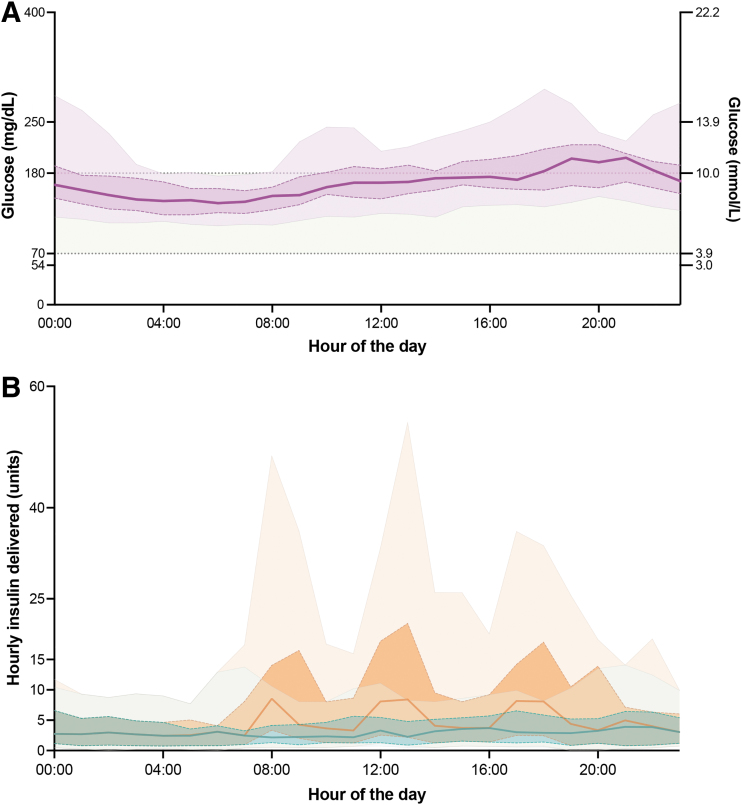
Hourly overview of glycemic control and insulin delivery with AID. **(A)** This panel illustrates the compiled 24-h glucose profiles from 16 selected patient hospitalizations that were eligible for glycemic analysis. The median glucose level is represented by a solid line, while the interquartile range is highlighted by a darker shaded region. The 5–95 percentile range is shown as a lightly shaded area. For contextual reference, the target glucose range (70–180 mg/dL; 3.9–10 mmol/L) is displayed within a light green band. **(B)** This panel visualizes the aggregated hourly data on basal insulin (blue) and total insulin (orange) administration from the same 16 patient hospitalizations over a 24-h period. Median insulin delivery rates are indicated by solid lines, with the interquartile range represented by darker shaded regions. The 5–95 percentile range is outlined by the lighter shaded areas. AID, automated insulin delivery.

Figure 4A shows 24-h glucose profiles with interquartile and 5–95 percentile ranges during treatment with AID, and Figure 4B shows the associated hourly median basal insulin infusion rate. Basal insulin delivery peaked at 21:00 h and nadired at 08:00 h. Mean daily basal insulin delivery was 0.21 ± 0.12 units/kg/day (range 0.06–0.48 units/kg/day) and mean TDD was 0.41 ± 0.21 units/kg/day (range 0.1–0.85 units/kg/day).

Cumulative time in automated mode totaled 2205 h, during which insulin delivery was suspended for 565 h (23%) and limited mode was active for 11 h (<1%) for the 18 participants. The various glucose target levels, including 150 mg/dL (8.3 mmol/L), 140 mg/dL (7.8 mmol/L), 130 mg/dL (7.2 mmol/L), 120 mg/dL (6.7 mmol/L), and 110 mg/dL (6.1 mmol/L), represent the major tuning parameters in the system's automated mode, and were utilized for 8.1%, 11.5%, 11.6%, 59.5%, and 19.2% of the time, respectively. In addition, Activity Feature, which increases the target to 8.3 mmol/L *and* reduces insulin delivery by ∼50%, was activated for 117 h (5%). Median percent time with insulin suspended per hospitalization was 20% (IQR 0%, 41%). There were no instances of DKA or severe hypoglycemia.

The 18 participants used a total of 25 sensors (6 participants required 7 sensor replacements, 4 of which were due to issues with validation/calibration, 2 due to operations/procedures requiring device removal, and 1 due to bleeding at insertion site). One additional sensor was removed due to issues with the device function. A total of 597 paired CGM and reference capillary glucose values were collected using 20 sensors (excluding the 4 mentioned above with validation/calibration issues and the 1 removed due to bleeding). Mean absolute relative difference (MARD) across the 20 sensors was 12.3%. The proportion of CGM values within ±15 or 20% of reference CBG values for levels ≥70 mg/dL (3.9 mmol/L) and ±15 or ±20 mg/dL (0.8 or 1.1 mmol/L) for CBG levels <70 mg/dL (i.e., 15/15% and 20/20% agreement) was 72.5% and 83.8%, respectively.

The percentage of paired values falling within Clarke Zone A or B was 99.5% (Supplementary S3).^25^ Data regarding sensor validation success rates, exchanges, and calibration frequency are reported in Supplementary S4.

Two participants (11%), discharged earlier than anticipated, did not complete the questionnaire assessing participant satisfaction. Among the 16 participants who completed the questionnaire, all of them (100%) either agreed (50%) or strongly agreed (50%) with the statement, “Overall, I liked using the Omnipod 5/Horizon system to treat my blood sugar in the hospital.” Fifteen (94%) indicated they would want to use the system at home. However, 6 (38%; 4 from phase 1 and 2 from phase 2) found the required number of CBG finger sticks burdensome, and 3 (19%) preferred to use only the CGM without the pod. None found the number of alarms burdensome. In the free response questions, eight participants liked removal of the need for syringe insulin injections, two participants wanted a longer duration of pod life (>3 days) between exchanges, three wished for the removal of carbohydrate counting, and two suggested removing the need for bolusing.

## Discussion

This study demonstrates a novel approach to hospital glycemic management with AID. The use of remote real-time CGM and a disposable, tubeless patch-pump with remote insulin delivery and monitoring capabilities was feasible, with a median proportion of time spent in automated mode of 95% (IQR 92%, 98%), and effective, with a mean TIR of 68% ± 16% and minimal time spent in hypoglycemia. Importantly, these results were observed among a diverse and relevant population of hospitalized adults with T1D and T2D, full-dose insulin requirements, active clinical conditions, steroid use, long hospitalizations, and a range of prior home glucose control by HbA1c.

The COVID-19 pandemic sparked interest in technologies featuring remote control and monitoring capabilities. To care for patients in isolation, observational studies explored the feasibility of using remote monitoring.^10–12^ Moreover, two of four recent clinical trials utilizing real-time CGM with remote monitoring to detect hypoglycemia and to guide insulin therapy demonstrated that this approach may reduce hypoglycemia incidence in high-risk patients,^13,14^ but has minimal impact on overall glycemic control (Table 3).^13–16^ The present study suggests incorporating AID with remote operability and components that are physically compatible with the inpatient environment to real-time remote CGM is feasible and has the potential to enhance glycemic control in hospitals, with minimal risk of hypoglycemia.

**Table 3.** AIDING Feasibility Results in the Context of Select Recent Inpatient Studies with Continuous Glucose Monitoring Data

	*N*	Mean glucose			TIR (70–180 mg/dL; 3.9–10.0 mmol/L)			TBR (<70 mg/dL; <3.9 mmol/L)		
		Interv, mg/dL mmol/L	Ctrl, mg/dL mmol/L	*P*	Interv, %	Ctrl, %	*P*	Interv, %	Ctrl, %	*P*
MDI										
Fortmann et al.^15^ (MDI: rtCGM vs. POC)	110	192 ± 39 10.7 ± 2.2	212 ± 46 11.8 ± 2.6	0.01	25 (12–43)	20 (3–40)	0.15	0.0 (0.0–0.0)	0.0 (0.0–0.0)	0.27
Singh et al.^13^ (MDI: rtCGM vs. POC)	72	183^a^ 10.2^a^	180^a^ 10.0^a^	0.69	59 (53–67)	55 (48–62)	0.39	0.4 (0.2–0.9)	1.9 (1.3–2.8)	<0.01
Klarskov et al.^16^ (MDI: rtCGM vs. POC)	64	200 ± 56 11.1 ± 3.1	194 ± 72 10.8 ± 4.0	0.37	46 (15–75)	68 (14–85)	0.37	0.0 (0.0–0.0)	0.0 (0.0–0.5)	0.60
Spanakis et al.^14^ (MDI: rtCGM vs. POC)	185	183 ± 40 10.2 ± 2.2	187 ± 39 10.4 ± 2.2	0.36	55 ± 28	49 ± 24	0.14	0.7 ± 2.2	2.2 ± 5.9	0.43
AID										
Thabit et al.^19^ (AID vs. MDI)	40	160 ± 31 8.9 ± 1.7	182 ± 38 10.1 ± 2.1	0.07	60 ± 19^b^	38 ± 17^b^	<0.01	0.0 (0.0–0.4)^c^	0.0 (0.0–2.7)^c^	0.35
Bally et al.^17^ (AID vs. MDI)	136	154 ± 29 8.6 ± 1.6	188 ± 43 10.4 ± 2.4	<0.01	66 ± 17^b^	42 ± 17^b^	<0.01	0.5 (0.0–1.1)	0.0 (0.0–1.8)	0.13
Boughton et al.^26^ (AID vs. MDI)	43	153 ± 22 8.5 ± 1.2	205 ± 61 11.4 ± 3.4	<0.01	68 ± 16^b^	36 ± 27^b^	<0.01	0.5 (0.0–1.5)	0.5 (0.0–2.9)	0.74
Herzig et al.^18^ (AID vs. MDI)^d^	45	144 ± 13 8.0 ± 0.7	169 ± 45 9.4 ± 2.5	0.03	84 ± 9	64 ± 25	<0.01	0.2 (0.0–0.6)	0.0 (0.0–0.6)	0.42
Boughton et al.^20^ (AID only)^e^	32	192 ± 34 10.7 ± 1.9	NA^f^		53 ± 18	NA^f^		0.4 (0.0–0.9)	NA^f^	
AIDING Feasibility, 2023^g^ (AID only)	**16**	**167 ± 21** **9.3 ± 1.2**	NA^f^		**68 ± 16**	NA^f^		**0.0 (0.0–0.2)**	NA^f^	

Bolded values denote results from the present study.

^a^ No standard deviation was reported.

^b^ TIR is reported as 100–180 mg/dL (5.6–10 mmol/L). Percent time in 70–180 mg/dL (3–10 mmol/L) is not available.

^c^ TBR is reported as <63 mg/dL (<3.5 mmol/L). Percent time <70 mg/dL (<3.9 mmol/L) is not available.

^d^ All undergoing planned elective surgery anticipated to last ≥2 h.

^e^ Real-world use over 12 months.

^f^ Single-arm study without a control group.

^g^ Present study.

AID, automated insulin delivery; AIDING, Automated Insulin Delivery for INpatients with DysGlycemia; Ctrl, control, Interv, intervention; MDI, multiple daily injection (insulin); POC, point-of-care (capillary finger stick) glucose; rtCGM, real-time continuous glucose monitoring; TBR, time below range.

Indeed, the glycemic results observed in this study align with hospital studies conducted in Europe with the CamAPS HX algorithm in fully closed-loop configuration without meal announcements (Table 3).^17–19,26^ These studies have consistently demonstrated the effectiveness of the CamAPS system in improving glycemic control in the hospital. For instance, the initial study in patients with T2D achieved a TIR of 59.8% ± 18.7% in the closed-loop group, compared with 38.1% ± 16.7% in the control group.^19^ These findings were replicated in a larger trial conducted by Bally et al. in which closed-loop TIR was 24.3% ± 2.9% (95% confidence interval 18.6–30.0) higher than in controls.^17^ Consistent outcomes were also seen in patients receiving medical nutrition therapy and during the perioperative period.^18,26^ However, these studies did not systematically involve nursing staff, address CGM accuracy concerns, or incorporate EHR documentation. Efforts to implement CamAPS HX in real-world settings are underway.^20^

The reliability of CGM is crucial for the feasibility of AID in the hospital. A pooled analysis of CGM study data using the G6 was promising, indicating that CGM use is reliable for hospitalized patients with diabetes, with an overall MARD of 12.3%.^27^ This and other studies have also highlighted how clinical conditions and situations associated with hospitalization can affect CGM reliability, such as severe anemia, hypoglycemia, mechanical interferences, and perioperative issues.^22,27^ Therefore, this feasibility study used a hybrid CGM-CBG glucose monitoring validation strategy, initially introduced in the ICU to reduce nursing staff burdens when caring for patients with COVID-19, which allowed for real-time accuracy verification and sensor calibration to address inaccuracies.^10,11^ Calibrations were not uncommon in this study (median 1 per participant), as noted in Supplementary S4. Accordingly, the ability to calibrate CGM sensors may be an important factor in determining their appropriateness for inpatient use.

This study has several important limitations. First, it was a single-arm design, and further exploration in a larger randomized trial with a control arm is necessary to confirm these findings and draw conclusions about the magnitude of the impact and the generalizability of its efficacy and safety. In addition, given the small sample size, a statistical subanalysis by subgroups (e.g., type of diabetes) was not possible. Second, although investigators made efforts to establish an infrastructure for nursing staff to manage the system, the resources invested in managing participants in this study may not yet be scalable. Future studies should incorporate implementation and scalability aspects into the trial design from the outset.

## Conclusions

This study represents an important initial step in evaluating the feasibility of using an AID system equipped with a tubeless and disposable patch-pump and remote monitoring protocol (insulin delivery and glucose monitoring) in a hospital environment. Furthermore, it demonstrates that nursing staff can actively perform remote insulin delivery, glucose monitoring, validation of sensor glucose values, and EHR documentation, opening opportunities to design trials that emphasize implementation aspects of care to promote the scalability of diabetes technology use in the hospital. Overall, these findings highlight the substantial promise that AID systems hold toward enhancing diabetes management in the hospital, including among patients not previously exposed to advanced diabetes technologies.

## Supplementary Material

Supplemental data
