# Supplementary material for: Automated Insulin Delivery with Remote Real-Time Continuous Glucose Monitoring for Hospitalized Patients with Diabetes: A Multicenter, Single-Arm, Feasibility Trial
Source: Diabetes Technol Ther. 2023 Oct 12;25(10):677–88. doi: 10.1089/dia.2023.0304 (PMC10611957; doi:10.1089/dia.2023.0304)
Supplement: Supplemental data [file Suppl_Data.pdf]

Online-only Supplemental Material

Automated Insulin Delivery with Remote Real-Time Continuous Glucose Monitoring for Hospitalized Patients with Diabetes: A Multi-Center, Single-arm, Feasibility Trial

Georgia M. Davis, MD*[1]; Michael S. Hughes, MD*[2]; Sue A. Brown, MD[3]; Judy Sibayan, MPH[4]; M. Citlalli Perez-Guzman, MD[1]; Meaghan Stumpf, MD[3]; Zachary Thompson, MS[4]; Marina Basina, MD[2]; Ronak M. Patel, MD[3]; Joi Hester, MD[1]; Amalia Abraham, BS[1]; Trang Ly, MBBS, PhD[5]; Cherie Chaney, FNP[3]; Marilyn Tan, MD[2]; Liana Hsu, BS[6]; Craig Kollman PhD[4]; Roy W. Beck, MD, PhD[4]; Rayhan Lal, MD[2,6]; Bruce Buckingham, MD[6]; Francisco J. Pasquel, MD, MPH[1]

* Co-first authors

1. Division of Endocrinology, Metabolism, and Lipids, Emory University School of Medicine, Atlanta, GA
2. Division of Endocrinology, Gerontology and Metabolism, Department of Medicine, Stanford University, Stanford, CA
3. Division of Endocrinology, Center for Diabetes Technology, University of Virginia, Charlottesville, VA
4. Jaeb Center for Health Research, Tampa, Florida
5. Insulet Corporation, Acton, MA
6. Division of Pediatric Endocrinology, Department of Pediatrics, Stanford University, Stanford, CA

Table of Contents

Supplement S1 | Patient Questionnaire (given at study completion) **2**

Supplement S2 | Individual Patient-level Hospitalization Data from the AIDING Feasibility Study **3**

Supplement S3 | CGM Accuracy as Compared to Capillary Blood Glucose During the AIDING Study **5**

Supplement S4 | CGM Utilization Requirements **6**

Supplement S5 | Full Study Protocol **7**

Supplement S1 | Patient Questionnaire (given at study completion)

1) Overall, I liked using the Omnipod 5/Horizon system to treat my blood sugar in the hospital.

    ☐ 5-Strongly Agree
    ☐ 4-Agree
    ☐ 3-Neither Agree nor Disagree
    ☐ 2-Disagree
    ☐ 1-Strongly Disagree

2) If you have diabetes, would you want to use this system to manage your diabetes at home?

    ☐ Yes
    ☐ No
    ☐ Not applicable

3) Did you find the number of alarms burdensome?

    ☐ Yes
    ☐ No

4) Did you find the number of fingersticks burdensome?

    ☐ Yes
    ☐ No

5) Would you prefer to use the ONLY Dexcom sensor WITHOUT the pump (pod)?

    ☐ Yes
    ☐ No

6) What did you like about using the Omnipod 5/Horizon System?

    [Free response]

7) What did you dislike about using the Omnipod 5/Horizon System?

    [Free response]

8) What recommendation(s) can you make to help us make the system better or easier to use?

    [Free response]

A.

B.

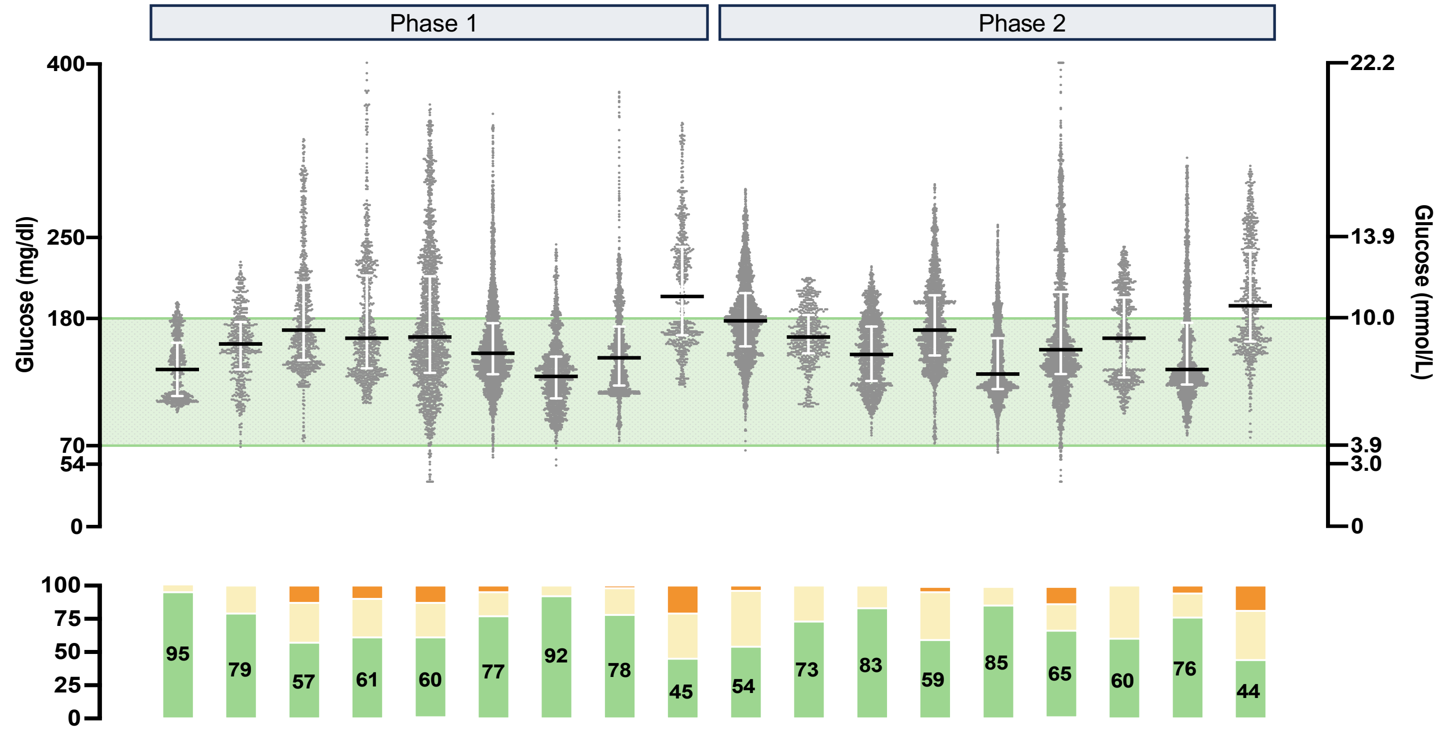

C.

Participant ID	1	2 [a]	3	4	5	6	7	8	9	10	11 [a]	12	13	14	15	16	17	18
**Age (years)**	34	67	65	83	65	66	60	61	64	48	45	81	59	51	32	47	65	67
**Gender**	M	M	F	F	M	M	M	M	M	F	M	M	M	M	M	M	F	F
**Race/Ethnicity**	Black /AA	White	Black /AA	Black /AA	Black /AA	White	Hisp Asian	White	White	Black /AA	Black /AA	White	White	White	Hisp	White	White	Black /AA
**Reason for admission**	Sepsis (DM foot)	Chest pain	Chest pain	Hip fract	Heart fail	Afib	Infxn (PNA)	Resp fail & lung transp	LFT elev post ERCP	Infxn (UTI)	Infxn (OM)	Infxn (DM foot)	Infxn (groin hema-toma)	Resp fail & lung transp	Low BG	Phle-bitis	ACS	Heart fail
**Diabetes type**	T2	T2	T2	T2	T2	T2	T2	T2	T2	T1	T2	T2	T2	T2	T1	T2	T2	T2
**HbA1c**																		
%	11	8.2	7.5	8.4	7.1	6.7	8.3	6.4	6.3	7.4	10.8	7	9.7	7.7	9.7	7.5	8.6	8.9
mmol/mol	96.7	66.1	58.5	68.3	54.1	49.7	67.2	46.4	45.4	57.4	94.5	53	82.5	60.7	82.5	58.5	70.5	73.8
**Glu mean ±SD**																		
mg/dl	138 ±26	157 ±31	184 ±52	181 ±56	177 ±64	159 ±43	132 ±31	153 ±42	208 ±54	181 ±36	165 ±25	151 ±29	176 ±38	142 ±33	171 ±60	165 ±38	154 ±48	199 ±48
mmol/L	7.7 ±1.4	8.7 ±1.7	10.2 ±2.9	10.0 ±3.1	9.8 ±3.5	8.8 ±2.4	7.3 ±1.7	8.5 ±2.3	11.5 ±3.0	10.0 ±2.0	9.1 ±1.4	8.4 ±1.6	9.7 ±2.1	7.9 ±1.8	9.5 ±3.3	9.2 ±2.1	8.6 ±2.7	11.0 ±2.7
**Total days**	2.4	1.7	2.9	2.7	6.2	9.8	5.8	5.9	2.1	9.5	1.9	5.7	7.5	9.9	9.6	2.6	7.5	2.3
**% Time automated**	71	60	94	97	94	98	98	96	80	92	96	96	98	98	99	92	93	93
**%TAR_{VH}**	0	0	13	10	13	5	0	2	21	4	0	0	4	0	13	0	6	19
**%TAR_{H}**	6	21	30	29	26	18	8	20	34	42	27	17	36	14	20	40	18	37
**% TIR**	95	79	57	61	60	77	92	78	45	54	73	83	59	85	65	60	76	44
**%TBR_{L}**	0	0	0	0	1	0	0	0	0	0	0	0	0	0	1	0	0	0
**% TBR_{VL}**	0	0	0	0	<1	0	<1	0	0	0	0	0	0	0	<1	0	0	0

Supplement S2 | Individual Patient-level Hospitalization Data from the AIDING Feasibility Study

The figure is organized into three vertically-aligned panels (A-C). Each column represents a individual study participant, ordered and numbered sequentially in order of trial participation. The two trial phases are additionally depicted above the panels (Phase 1 necessitated six daily CGM validations, while Phase 2 required four). **A.)** Each clinical histogram (or non-smoothed raw violin plot) illustrates the complete set of individual CGM values analyzed (depicted in grey) for each participant starting from the time of automated mode initiation until discontinuation. The median is visually represented by a horizontal black line, while the interquartile range is indicated by the white bars. **B.)** Stacked bars visually represent the percentage of time the corresponding participant spent in each glycemic range (color scheme corresponds to the table in panel C), providing an overview of the distribution of glycemic levels. **C)** The table presents essential demographic information and

glycemic outcomes for each participant, offering a comprehensive view of their characteristics and glucose management results.

a. *Not included in endpoint analyses due to lack of prespecified requirement for >48 hours CGM data.*

Abbreviations: AA – African American, CGM – continuous glucose monitor, DM – diabetes mellitus, Glu – glucose, HbA1c – hemoglobin A1c, T1 – type 1 diabetes, T2 – type 2 diabetes, TAR_H *– time above range-high (Level 1 TAR,[27] 181-250 mg/dl, 10.1-13.9 mmol/L),* TAR_{VH} *– time above range-very high (Level 2 TAR,[27] >250 mg/dl, >13.9 mmol/L),* TBR_L *– time below range-low (Level 1 TBR,[27] 54-70 mg/dl, 3.0-3.9 mmol/L),* TBR_{VL} *– time below range-very low (Level 2 TBR,[27] <54 mg/dl, <3.0 mmol/L), TIR – time in range (70-180 mg/dl, 3.9-10 mmol/L).*

A. Absolute Relative Difference

Mean (MARD)	12.3%
Median	9.3%

**B. Agreement**	**N**	**%**
±%30/30	547	91.6%
±%20/20	500	83.8%
±%15/15	433	72.5%
±%10/10	315	52.8%

C. Clarke Error Grid

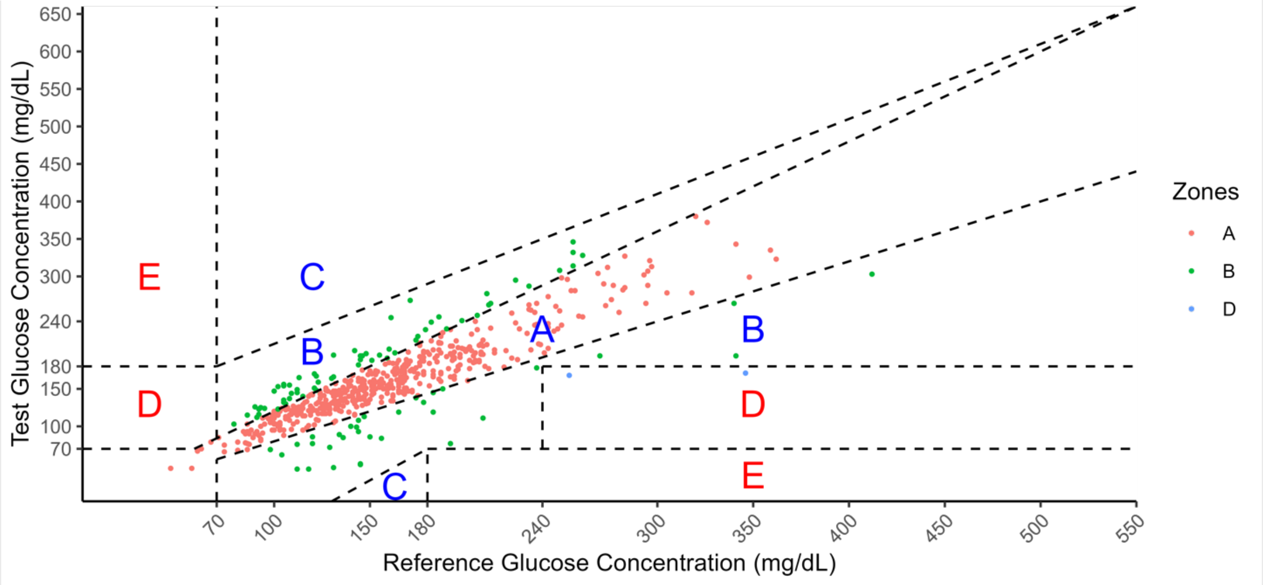

**D. Clarke Zone**[a]	**N**	**%**
Zone A	500	83.8%
Zone B	95	15.9%
Zone C	0	0.0%
Zone D	2	<1%
Zone E	0	0.0%

Supplement S3 | CGM Accuracy as Compared to Capillary Blood Glucose During the AIDING Study

Accuracy of the CGM was assessed as compared to simultaneous fingerstick capillary blood glucose (CBG) values. Data represent the 597 paired CGM and CBG values generated in the study from N = 18 patients. Of note, these values were compared real-time using the ±%20/20 criterion for accuracy assessment and as-needed calibrations, as described elsewhere. **A.)** Mean absolute relative difference (MARD) and median absolute relative difference between CGM and reference. **B.)** Agreement between CGM value and reference: Percent of CGM values within ±X% of CBG value (if ≥70 mg/dl or ≥3.9 mmol/L) or within ±X mg/dL of CBG (if <70 mg/dl or <3.9 mmol/L). **C.)** Clarke Error Grid. **D.)** Number and percent of paired values falling into each Clarke zone.

a. Clarke Zones are defined as follows: Zone A – CGM values within 20% of reference; Zone B – CGM values that are more 20% outside the reference but would not lead to an inappropriate treatment; Zone C – CGM values leading to unnecessary treatment; Zone D – CGM values that have potentially dangerous failure to detect hypoglycemia or hyperglycemia; Zone E – CGM values that would reverse exchange treatment of hypoglycemia for hyperglycemia and vice versa[28]

Supplement S4 | CGM Utilization Requirements

	Overall	Phase 1	Phase 2
Per participant	N=18	N=9	N=9
Percent time with active CGM data			
Mean ± SD	97% ± 4%	95% ± 5%	99% ± 1%
Median (IQR)	99% (96%, 100%)	96% (93%, 99%)	99% (99%, 100%)
Range	86% to 100%	86% to 100%	97% to 100%
Percent of scheduled validations that were successful			
Mean ± SD	85% ± 14%	85% ± 16%	85% ± 14%
Median (IQR)	86% (80%, 97%)	86% (80%, 97%)	86% (80%, 94%)
Range	50% to 100%	50% to 100%	60% to 100%
Number of calibrations			
Median (IQR)	1 (0, 3)	1 (1, 3)	1 (0, 2)
Range	0 to 4	0 to 4	0 to 4
None	5 (28%)	2 (22%)	3 (33%)
≥1	13 (72%)	7 (78%)	6 (67%)
≥2	6 (33%)	3 (33%)	3 (33%%)
≥3	5 (28%)	3 (33%)	2 (22%)
≥4	2 (11%)	1 (11%)	1 (11%)
Number of sensors – N	25	13	12
Sensors requiring early replacement – N (%)	7 (28%)	4 (31%)	3 (25%)

Supplement S5 | Full Study Protocol

Signature Page

Automated Insulin Delivery for INpatients with DysGlycemia (AIDING) Feasibility Study

IDE Sponsor: Francisco J. Pasquel, MD, MPH

Version Number: 2.3

28 JUN 2021

**Clinical Lead Investigator**	
**Name, degree**	Francisco J Pasquel, MD, MPH
**Signature/Date**	
**Medical Monitor**	
**Name, degree**	Roy W. Beck, MD, PhD
**Signature/Date**	
**Study supporter**	Insulet Corporation
**Name, degree**	Trang Ly, MBBS, FRACP, PhD
**Signature/Date**	

Automated Insulin Delivery for INpatients with DysGlycemia (AIDING) Feasibility Study

IDE Sponsor: Francisco J Pasquel, MD, MPH

Funded by: Insulet Corporation

Version Number: 2.3

28 JUN 2021

KEY ROLES

**Principal Investigator, Coordinating Site**	
**Name, degree**	Francisco J Pasquel, MD, MPH
**Title**	Assistant Professor
**Institution Name**	Emory University School of Medicine
**Medical Monitor**	
**Name, degree**	Roy W. Beck, MD, PhD
**Title**	Executive Director
**Institution Name**	Jaeb Center for Health Research
**Study supporter**	
**Name, degree**	Trang Ly, MBBS, FRACP, PhD
**Title**	SVP, Clinical and Medical Director
**Institution Name**	Insulet Corporation
**Site Principal Investigator**	
**Name, degree**	Bruce Buckingham, MD
**Title**	Professor
**Institution Name**	Stanford University School of Medicine
**Site Principal Investigator**	
**Name, degree**	Sue A. Brown, MD
**Title**	Associate Professor
**Institution Name**	University of Virginia School of Medicine

TABLE OF CONTENTS

CHAPTER 1: BACKGROUND INFORMATION 15
1.1 Introduction 15
1.2 Rationale 16
1.2.1 Inpatient diabetes management and glycemic control targets 16
1.2.2 Hybrid closed-loop insulin therapy 18
1.3 Preliminary Data 22
1.4 Potential Risks and Benefits of the [Investigational Device] 25
1.4.1 Known Potential Risks 25
1.4.1.1 Hyperglycemia and Hypoglycemia Risks 25
1.4.1.2 Other risks 26
1.4.2 Known Potential Benefits 26
1.4.3 Risk Assessment 26
1.5 General Considerations 27

CHAPTER 2: STUDY ENROLLMENT AND SCREENING 28
2.1 Participant Recruitment and Enrollment 28
2.2 Informed Consent and Authorization Procedures 28
2.3 Participant Eligibility 29
2.3.1 Inclusion Criteria 29
2.3.2 Participant Exclusion Criteria 29
2.4 Screening Procedures 29
2.5 Data Collection and Testing 30
2.5.1 Daily data recording 30

CHAPTER 3: STUDY PROCEDURES 30
3.1 Omnipod 5/Horizon automated insulin delivery system 30
3.1.1 Dexcom CGM validation 32
3.1.2 Ongoing glucose monitoring 33
3.1.3 Insulin Delivery 33
3.2 Unit-based monitoring 34
3.3 Remote monitoring by research staff 35
3.4 Interruption of HCL Therapy 35
3.5 Discontinuation of HCL/AID 36

CHAPTER 4: STUDY DEVICES 37
4.1 Description of the Investigational Device 37
4.1.1 Omnipod5/Horizon™ automated insulin delivery system 39

4.1.2 Continuous Glucose Monitoring 40
4.1.3 Horizon Data Portal 40
4.1.4 Blood Glucose Meter and Strips 40
4.1.5 Study Device Accountability Procedures 41
4.2 Safety Measures 41
4.2.1 CGM verification of validation 41
4.2.2 Pump Failure 41
4.2.3 Hypoglycemia Threshold Alarm and Safety Protocol 41
4.2.4 Hyperglycemia Threshold Alarm and Safety Protocol 41

CHAPTER 5: TESTING PROCEDURES AND QUESTIONNAIRES 42
5.1 Laboratory Testing 42
5.2 Surveys and Questionnaires 42

CHAPTER 6: UNANTICIPATED PROBLEM, ADVERSE EVENT, AND DEVICE ISSUE REPORTING 43
6.1 Unanticipated Problems 43
6.2 Adverse Events 43
6.2.1 Definitions 43
6.2.2 Reportable Adverse Events 44
6.2.3 Hypoglycemic Events 45
6.2.4 Hyperglycemic/Ketotic Events 45
6.2.5 Relationship of Adverse Event to Study Investigational Device 45
6.2.6 Severity (Intensity) of Adverse Events 46
6.2.7 Expectedness 46
6.2.8 Coding of Adverse Events 47
6.2.9 Outcome of Adverse Events 47
6.3 Reportable Device Issues 47
6.4 Timing of Event Reporting 48
6.5 Safety Oversight 48
6.6 Stopping Criteria 49
6.6.1 Participant Discontinuation of Study Device 49
6.6.2 Criteria for Suspending or Stopping Overall Study 50

CHAPTER 7: MISCELLANEOUS CONSIDERATIONS 51
7.1 Drugs Used as Part of the Protocol 51
7.2 Collection of Medical Conditions and Medications 51
7.3 Prohibited Medications, Devices, Treatments, and Procedures 51
7.4 Precautionary Medications, Treatments, and Procedures 52

7.5 Prophylactic Medications, Treatments, and Procedures ... 52
7.6 Rescue Medications, Treatments, and Procedures ... 52
7.7 Pregnancy Reporting ... 52
7.8 Participant Compensation ... 52
7.9 Participant Withdrawal ... 52
7.10 Confidentiality ... 52

CHAPTER 8: STATISTICAL CONSIDERATIONS ... 53
8.1 Statistical and Analytical Plans ... 53
8.2 Statistical Hypotheses ... 53
8.3 Sample Size ... 53
8.4 Outcome Measures ... 53
8.5 Analysis Dataset ... 54
8.6 Analysis of the Primary Efficacy Endpoint(s) ... 54
8.7 Analysis of the Secondary Endpoints ... 54
8.8 Safety Analyses ... 54
8.9 Intervention Adherence ... 55
8.10 Protocol Adherence and Retention ... 55
8.11 Baseline Descriptive Statistics ... 55
8.12 Device Issues ... 55
8.13 Planned Interim Analyses ... 55
8.14 Sub-Group Analyses ... 56
8.15 Multiple Comparison/Multiplicity ... 56
8.16 Exploratory Analyses ... 56
8.17 Additional Tabulations and Analyses ... 56

CHAPTER 9: DATA COLLECTION AND MONITORING ... 57
9.1 Case Report Forms and Other Data Collection ... 57
9.2 Study Records Retention ... 57
9.3 Quality Assurance and Monitoring ... 57
9.4 Protocol Deviations ... 58

CHAPTER 10: ETHICS/PROTECTION OF HUMAN PARTICIPANTS ... 59
10.1 Ethical Standard ... 59
10.2 Institutional Review Boards ... 59
10.3 Informed Consent Process ... 59
10.3.1 Consent Procedures and Documentation ... 59
10.3.2 Participant and Data Confidentiality ... 59

10.3.3 Future Use of Stored Specimens and Data 60

CHAPTER 11: REFERENCES 61

128 LIST OF ABBREVIATIONS

ABBREVIATION	DEFINITION
ACE	Alternate Controller Enabled
ADE	Adverse Device Effect
AE	Adverse Event
AID	Automated Insulin Delivery
ARD	Absolute Relative Difference
BG	Blood Glucose
BMI	Body Mass Index
CEG	Clarke Error Grid
CGM	Continuous Glucose Monitoring
CI	Confidence Interval
COVID-19	Coronavirus-2019
CRF	Case Report Form
CRP	C-Reactive Protein
CV	Coefficient of Variation
DIC	Disseminated Intravascular Coagulation
DKA	Diabetic Ketoacidosis
DM	Diabetes Mellitus
eGFR	Estimated Glomerular Filtration Rate
EGV	Estimated Glucose Value
EHR	Electronic Health Record
EKG	Electrocardiogram
FDA	Food and Drug Administration
GCP	Good Clinical Practice
GV	Glycemic Variability
HbA1c	Hemoglobin A1c
HCW	Healthcare Worker
HDP	Horizon Data Portal
ICR	Insulin-to-Carbohydrate Ratio
ICU	Intensive Care Unit
IDE	Investigational Device Exemption
IL-6	Interleukin-6
IRB	Institutional Review Board
ISF	Insulin Sensitivity Factor
IV	Intravenous
MARD	Mean Absolute Relative Difference
MPC	Model Predictive Control
PDM	Personal Diabetes Manager
POC	Point-of-Care
PPE	Personal Protective Equipment
PUI	Person Under Investigation
QA	Quality Assurance
QC	Quality Control

ABBREVIATION	DEFINITION
RBM	Risk-Based Monitoring
RCT	Randomized Controlled/Clinical Trial
SAE	Severe Adverse Event
SD	Standard Deviation
T1	Type 1
T2	Type 2
TAR	Time Above Range
TBI	Total Basal Insulin
TBR	Time Below Range
TDD	Total Daily Dose
TDI	Total Daily Insulin
TIR	Time In Range
UADE	Unanticipated Adverse Device Effect

SITE PRINCIPAL INVESTIGATOR STATEMENT OF COMPLIANCE

Protocol Title: **Automated Insulin Delivery for Inpatients with Dysglycemia (AIDING) Feasibility Study**

Protocol Version/Date: **v. 2.2 / 23 NOV 2020**

I have read the protocol specified above. In my formal capacity as a Site Principal Investigator, my duties include ensuring the safety of the study participants enrolled under my supervision and providing the Sponsor with complete and timely information, as outlined in the protocol. It is understood that all information pertaining to the study will be held strictly confidential and that this confidentiality requirement applies to all study staff at this site.

This trial will be carried out in accordance with ICH E6 Good Clinical Practice (GCP) and as required by the following (use applicable regulations depending on study location and sponsor requirements; examples follow): United States (US) Code of Federal Regulations (CFR) applicable to clinical studies (45 CFR Part 46, 21 CFR Part 50, 21 CFR Part 56, 21 CFR Part 312, and/or 21 CFR Part 812).

For the foreign sites, the trial will be carried out in accordance with ICH E6 Good Clinical Practice (GCP), the Declaration of Helsinki, and specific regulations applicable to the countries in which the trial will be conducted.

As the Principal Investigator, I will assure that no deviation from, or changes to the protocol will take place without prior agreement from the sponsor and documented approval from the Institutional Review Board (IRB), or other approved Ethics Committee, except where necessary to eliminate an immediate hazard(s) to the trial participants.

All key personnel (all individuals responsible for the design and conduct of this trial) have completed Human Participants Protection Training and Good Clinical Practice Training. Further, I agree to ensure that all staff members involved in the conduct of this study are informed about their obligations in meeting the above commitments.

Investigator's Signature ___________________________________ Date: _____ / _____ / _____

dd mmm yyyy

Investigator's Name: _____________________________________

Site Name/Number: ______________________________________

162 PROTOCOL SUMMARY

PARTICIPANT AREA	DESCRIPTION
**Title**	Automated Insulin Delivery for Inpatients with Dysglycemia (AIDING) Feasibility Study
**Précis**	This single-arm stepwise feasibility study will test initial deployment of hybrid closed-loop (HCL) automated insulin delivery (AID) using the Omnipod 5/Horizon HCL system with remote monitoring and device operation capabilities to hospitalized patients admitted to the general medical/surgical floor with diabetes (type 1 or type 2) requiring insulin therapy. All enrolled participants will be placed on HCL insulin therapy for 10 days or until hospital discharge (if less than 10 days) to determine functional operability of the system and its effect on glycemic control in the hospital setting. This study will generate preliminary data to inform the design of a large multi-institution randomized controlled trial to assess superiority of HCL compared to standard inpatient insulin therapy.
**Investigational Device**	The Omnipod 5/Horizon HCL system, consists of a disposable insulin infusion pump (or "pod"), a built-in model predictive control (MPC) insulin dosing algorithm, and a remote Personal Diabetes Manager (PDM) interface, that interact with a Dexcom G6 continuous glucose monitor (CGM) to automatically control insulin delivery based upon real-time glucose values. The PDM component also enables remote interaction with the system, including glucose monitoring as well as insulin dosing management and adjustments.
**Objectives**	Primary objective:<br>To investigate functional operability and implementation of the Omnipod 5/Horizon HCL system in the hospital setting.<br><br>Secondary objectives:<br>To describe glycemic control, device settings and insulin delivery parameters, and explore staff and patient perceptions associated with early use of the Omnipod 5/Horizon HCL system in the hospital setting.
**Study Design**	Pilot and Feasibility
**Number of Sites**	3
**Endpoint**	**Primary Endpoints:**<br>*Aim 1 (Operability):* Proportion of time spent in HCL after CGM sensor meets initial validation criteria [sensor glucose value is within ±20% of POC values (for glucose levels ≥70 mg/dL) or ±20 mg/dL for POC glucose values <70 mg/dL].<br><br>*Aim 2 (Glycemic control):* Percentage of time sensor glucose is within target glucose range [time-in-range (TIR)], defined as 70-180 mg/dL.<br><br>**Key Secondary Endpoints:**<br>*Aim 1.1 Operability (System function):*

PARTICIPANT AREA	DESCRIPTION
	- Time from enrollment to start of HCL therapy (after initial CGM validation)<br>- Percentage of time with CGM readings<br>- Percentage of CGM values meeting accuracy criteria for bolus/correction insulin dosing<br>- Number of CGM readings within %15/15 of POC readings and within %20/20 of POC readings with the cut point at 70 mg/dL<br><br>*Aim 1.2 Perception (qualitative analyses):*<br>- Patients survey and open-ended feedback<br>*Aim 2.1. Glycemic control:*<br>- Number of hypoglycemic (<70 mg/dL) and clinically important hypoglycemic (<54 mg/dL) episodes per patient and per patient-day<br>- Percent time below range (TBR, <70mg/dL)<br>- Percent time below range (<54 mg/dL)<br>- Percent time above range (TAR, >180 mg/dL)<br>- Percent time in severe hyperglycemia (>250 mg/dL)<br>- Coefficient of variation (CV), standard deviation (SD)<br>*Aim 2.2 HCL Settings:*<br>- Frequency of setting adjustments for clinically-important hypoglycemia (<54 mg/dL): Overall, to basal rate, to insulin carb ratio (ICR), to insulin sensitivity factor (ISF)<br>- Frequency of setting adjustments for prolonged hyperglycemia (>250 mg/dL for >1 hour): Overall, to basal rate, to ICR, to ISF<br>- Insulin requirements: Total daily insulin (TDI), Total daily basal insulin (TBI), Total daily bolus (meal and correction).<br>**Key Safety Outcomes:**<br>- Reportable hypoglycemia: defined as an event that required assistance of another person due to altered consciousness to actively administer carbohydrate, glucagon, or other resuscitative actions. This means that the participant was impaired cognitively to the point that the participant was unable to treat his or herself, was unable to verbalize his or her needs, was incoherent, disoriented, and/or combative, or experienced seizure or coma.<br>- Diabetic ketoacidosis
**Population**	**Key Inclusion Criteria:**<br>- Patients ≥18 years of age with insulin-treated T1 or T2DM admitted to general (non-intensive care) medical-surgical hospital service requiring inpatient insulin therapy.

PARTICIPANT AREA	DESCRIPTION
	**Key Exclusion Criteria:**<br>- Patients admitted the ICU or anticipated to require ICU transfer<br>- Anticipated length of hospital stay <48 hours.<br>- Evidence of hyperglycemic crises (diabetic ketoacidosis or hyperosmolar hyperglycemic state) at enrollment<br>- Severely impaired renal function (eGFR < 30 ml/min/1.73m^2) or clinically significant liver failure<br>- Severe anemia with hemoglobin <7 g/dL<br>- Evidence of hemodynamic instability<br>- Hypoxia (SpO2 <95% on supplemental oxygen)<br>- Pre-admission or inpatient total daily insulin dose >100 units<br>- Mental condition rendering the participant unable to consent or answer questionnaires<br>- Pregnant or breast-feeding at time of enrollment<br>- Unable or unwilling to use rapid-acting insulin analogs (Humalog, Admelog, Novolog or Apidra) during the study<br>- Use of hydroxyurea or high-dose ascorbic acid (>1g/day)<br>- COVID-19 infection or person under investigation (PUI) on isolation precautions
**Sample Size**	18 patients
**Phase**	Pilot, Feasibility
**Treatment Groups**	All enrolled participants will be placed on HCL insulin therapy for up to 10 days or until hospital discharge.
**Participant Duration**	Up to 10 days during inpatient stay

163

Overview of Study Design

This multicenter study testing the feasibility of AID (Omnipod 5/Horizon HCL system) in the hospital will be conducted across three sites [Emory, Stanford, University of Virginia (UVA)]. To ensure safety of participants with this new impatient glycemic control approach we will employ a stepwise enrollment plan with two phases (**Figure 1**). We will enroll patients ≥18 years of age with insulin-treated type 1 (T1) or type 2 (T2) DM requiring insulin therapy during hospitalization admitted to the general medical/surgical floor (non-ICU). To have 9 subjects in each phase cohort who have used the system for at least 48 hours, we will enroll up to a total of 30 participants. All participants will be placed on AID for inpatient glucose management.

The first phase of the study will combine automated insulin delivery (AID) with intensified POC glucose monitoring (minimum of 6 POC glucose tests per day; n=9 subjects). During phase 2, AID will continue with less frequent POC glucose monitoring (approximately 4 times per day, standard-of-care; n=9 subjects) (**Figure 1**). IRB-approved informed consent will be obtained by study personnel designated at each site. Exclusion criteria include: **1)** patients requiring total daily insulin dose >100 units, **2)** patients admitted to ICU, **3)** patients anticipated to require <48 hours of admission, **4)** patients with evidence of hyperglycemic crises (diabetic ketoacidosis or hyperosmolar hyperglycemic state), **5)** patients with severely impaired renal function (eGFR < 30 ml/min/1.73m^2), **6)** clinically significant liver failure, **7)** severe anemia (hemoglobin <7 g/dL), **8)** hypoxia (SpO2 <95% on supplemental oxygen), **9)** evidence of hemodynamic instability, **10)** active or suspected COVID-19 infection, **11)** a mental condition rendering the participant unable to consent or answer questionnaires, **12)** pregnancy or breast feeding at time of enrollment, or **13)** use of hydroxyurea or high-dose ascorbic acid.

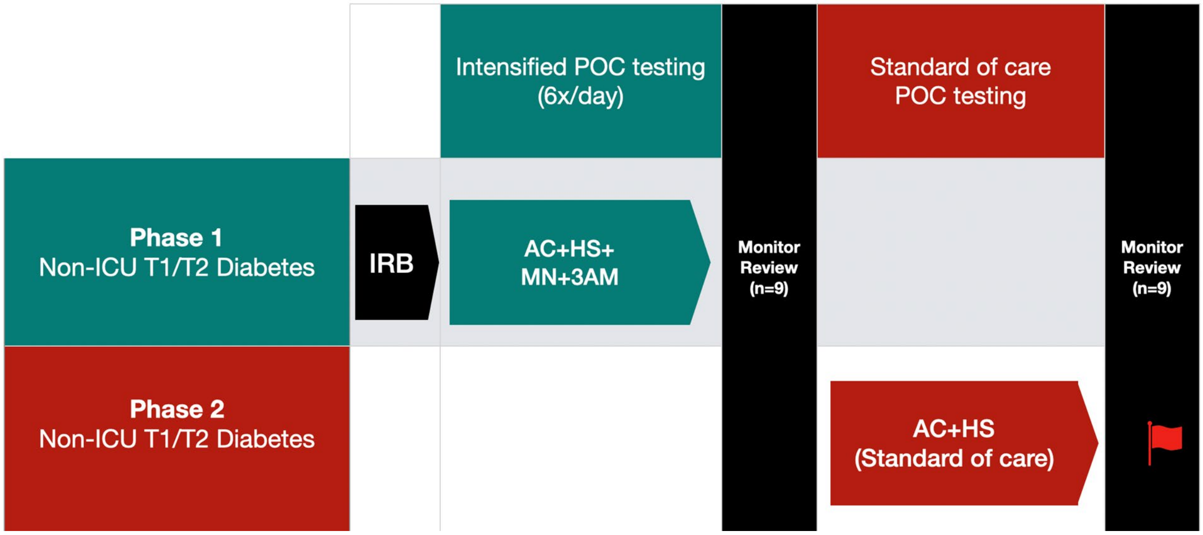

Figure 1. AIDING Feasibility Study Stepwise Enrollment Design. IRB: Institutional Review Board; POC: point-of-care testing; AC: Before meals; HS: bedtime; MN: midnight.

Chapter 1: Background Information

1.1 Introduction

The purpose of this study is to assess the safety and efficacy of using the Omnipod 5/Horizon automated insulin delivery (AID) system in improving glycemic control for hospitalized patients requiring subcutaneous insulin therapy. This is the first automated insulin delivery system which is well-suited for the hospital environment given its remote management capabilities with disposable components.

The coronavirus disease 2019 (COVID-19) pandemic has highlighted how accelerated use of technologies [e.g., telemedicine, e-consults, and remote monitoring] has helped healthcare systems adapt care delivery while minimizing exposure risk. The pandemic has also shed light on the bedside care burden associated with inpatient diabetes management in its current state. As more patients are admitted with COVID-19 infection, there is continued concern surrounding resource limitations, including PPE. These concerns have led to transformations in clinical care that focus on the preservation of scarce resources while continuing to provide the highest level of clinical care. Inpatient diabetes (DM) management is one realm of clinical care that has seen major changes in the setting of COVID-19, mostly due to the time- and labor-intensive efforts required to care for hospitalized patients with DM.[1] In the United States, it is estimated that about one in five patients with COVID-19 admitted to the hospital has DM.[2] The increasing proportion of hospitalized patients with DM and concurrent COVID-19 infection continues to highlight the importance of advancing inpatient diabetes care to balance the competing demands of frequent glucose monitoring and timely insulin administration with the bedside contact required for these efforts when PPE is scarce.

In Atlanta, a diagnosis of diabetes is associated with a threefold increase in the odds of hospitalizations.[3] Among those patients with COVID-19 hospitalized in Georgia (primarily metropolitan Atlanta) beginning in February, DM was documented in 39.7% of hospitalized with COVID-19,[4] and a recent report showed about 46% of critically ill patients had DM in COVID units in the Emory healthcare system.[5] Over 3500 cases of COVID-19 have been documented at Emory until the end of July 2020, including 883 patients with diabetes, of whom 114 expired (14%). At Stanford, of 2345 patients hospitalized with COVID-19 from March 1 to June 30, 2020, 25% (580) had diabetes. Among these 19% were intubated, and 2.6% of these patients died. At UVA, a sample of 141 patients hospitalized with COVID-19 showed 52% required treatment with insulin. We estimate at least 30% of patients with COVID-19 will have DM at the three centers.

The use of AID systems continues to grow in the outpatient setting among patients with both type 1 and type 2 diabetes. Preliminary data from AID trials with a single non-commercial European system (using an insulin pump with tubing) has shown significant improvements in glycemic control in diverse populations, without increasing the risk of hypoglycemia.[6-8] There is, however, no clinical trial data with the use of AID in the hospital in the US.

The AID system consists of the Omnipod 5/Horizon insulin infusion pump or “pod,” the Horizon Personal Diabetes Manager (PDM) and a Dexcom G6 CGM **(Figure 2)**. The system’s embedded model predictive control (MPC) algorithm utilizes CGM glucose values, rate of glucose change, and active insulin from previous boluses to make insulin dosing decisions. The algorithm has been tested in hundreds of patients ranging in age from 2 to 70 years old and has been stress tested with missed insulin boluses for meals, exercise, and insulin over-boluses for meals **(Table 1)**. The pod holds 200 units of insulin, the embedded algorithm, and directly receives Bluetooth glucose values from the G6 sensor. The pod is worn for three days and then disposed, thereby eliminating the possibility of cross-contamination between patients. The G6 CGM sensor lasts 10 days, while its transmitter lasts 3 months. Neither CGM component (sensor or transmitter) will be reused between patients. The system uses a dedicated smartphone [Horizon Personal Diabetes Manager (PDM)] to allow remote monitoring (via cellular data and Wi-Fi networks). Insulin boluses and adjustments to pump settings can be made from the smartphone if it is within Bluetooth distance of the pod (about 20-30 feet). We have tested this communication within the hospital setting and it has worked well, allowing remote bolusing and monitoring from outside of a patient's room (see preliminary data).

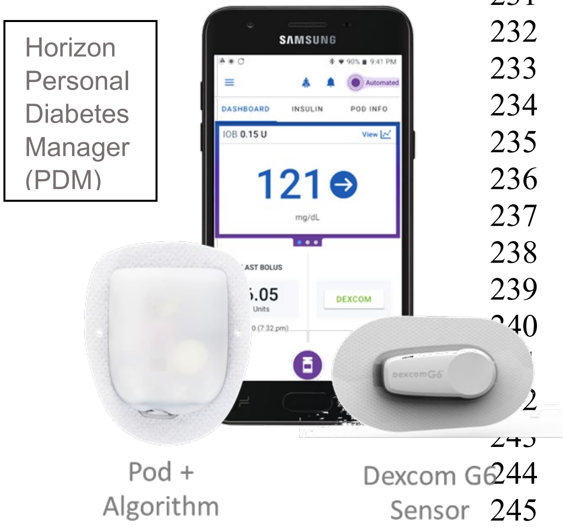

Figure 2. Omnipod5/Horizon AID system.

1.2 Rationale

1.2.1 Inpatient diabetes management and glycemic control targets

In the Leuven Surgical Trial, Van den Berghe et al. reported improvement in mortality and morbidity in adult ICU patients using a glucose target of 80-110 mg/dL.[9-12] This mobilized a drive to significantly reduce inpatient glucose levels. However, NICE-SUGAR, a large multicenter randomized controlled trial aiming to reproduce those findings, instead showed an increase in 90-day mortality in the intensive management group targeting euglycemia.[13] These results were later attributed to an increased rate of moderate to severe hypoglycemia seen among the group with more intensive glycemic targets (81-108 mg/dL).[14] Subsequently, to balance the important benefits of treating hyperglycemia against the increased risk of hypoglycemia in hospitalized patients, the American Diabetes Association (ADA) and American Association of Clinical Endocrinologists (AACE) recommended an inpatient target glucose range of 140-180 mg/dL. Similarly, the Society of Critical Care Medicine (SCCM) and the European Society of Intensive Care Medicine (ESICM) have also adopted a glucose goal ≤180 mg/dL in their collaborative Surviving Sepsis Campaign guidelines on the management of sepsis and septic shock. While these parameters provide the current standard directing inpatient glucose management, glycemic variability (GV) has also generated interest since several studies have shown an increased risk of mortality independent of mean glucose levels associated with increased GV.[15-17]

Consideration has been made as to whether strict glucose control can improve outcomes specifically in hospitalized patients with COVID-19. A multi-center retrospective analysis of 7,337 patients with COVID-19 in Hubei Province, China[18] reported an increased incidence of acute respiratory distress syndrome (ARDS), acute kidney injury, acute cardiac injury, disseminated intravascular coagulation (DIC), and all-cause in-hospital mortality among patients with pre-existing DM. As expected, these patients also had higher levels of acute inflammatory markers [C-reactive protein (CRP), procalcitonin, and D-dimer]. Yet, a sub-analysis of the cohort with DM revealed that in those with well-controlled glucose values (70-180 mg/dL), these endpoints and laboratory findings were all significantly reduced.[18] Similar findings were also reported by Sardu et al.[19] in a 28-day prospective study to assess the effect of intensive glycemic control on progression to severe COVID-19 disease (ICU admission, mechanical ventilation, or death). In this study of 59 patients admitted with moderate COVID-19 pneumonia, two subgroups were characterized based on the admission glucose >140 mg/dL (hyperglycemic), or <140 mg/dL. Of the hyperglycemic patients, 72% had a diagnosis of DM prior to admission. All 25 patients with hyperglycemia were asked to consent to intravenous (IV) insulin infusion if their glucose exceeded 180 mg/dL. Fifteen patients consented and were given IV insulin to target a glucose of 140-180 mg/dL, while the other 10 patients received standard basal-bolus subcutaneous insulin injections. The mean glucose during hospitalization for those receiving IV insulin therapy was 138 ± 33 mg/dL, and 192 ± 15 mg/dL for those not receiving IV insulin. Hyperglycemia and higher baseline levels of interleukin 6 (IL-6) and D-dimer were associated with progression to severe disease. However, of those who opted for IV insulin, only 33% progressed to severe disease, compared to 80% of those who did not. Additionally, those receiving IV insulin demonstrated much larger reductions in IL-6 and D-dimer levels at one- and two-week time points. These early studies suggest that improved glucose control may promote better clinical outcomes among patients with COVID-19.

However, use of continuous IV insulin infusion requires hourly interventions and significant oversight by healthcare providers. In non-ICU patients, basal-bolus insulin regimens continue to be the mainstay of therapy for patients with hyperglycemia in the hospital, relying heavily on the ability of healthcare workers (HCW) to perform frequent blood glucose monitoring and timely administration of multiple insulin injections to achieve glycemic control.[20] During COVID-19, related shortages in PPE and the healthcare workforce will undoubtedly have a negative impact on inpatient glycemic control and outcomes for these patients. Additionally, inpatient hyperglycemia itself may become increasingly challenging to mitigate, as rapidly evolving data has directed attention towards the use of high-dose steroids in the treatment of COVID-19.[21] Recent preliminary results from the "Randomised Evaluation of COVID-19 Therapy" (RECOVERY) trial showed a significant reduction in 28-day mortality among patients requiring supplemental oxygen (including non-invasive ventilation) or invasive mechanical ventilation treated with high-dose dexamethasone (6 mg daily).[22] Therefore, in light of the baseline prevalence and prognostic implications of pre-existing DM, early reports suggesting an association between glucose control and improved outcomes, and the now probable forthcoming increase in steroid usage among patients with COVID-19, maintaining glycemic control has become an integral part of inpatient COVID-19 management.

Advances in diabetes technology have changed the face of diabetes care in the outpatient setting through the use of CGM, insulin pump therapy and the integration of these two technologies to

provide AID.[23] Recent efforts have focused on moving these technologies to the inpatient setting to improve hospital diabetes management.[24] Our interest in this concept predates the pandemic, and we have previously reported our experience using CGM in the inpatient setting, including the implementation of a glucose telemetry system (NCT03877068).[25-28] Recently, this progress has been accelerated and has become available for more widespread clinical implementation during the COVID-19 pandemic. During the beginning of the pandemic, the US Food and Drug Administration (FDA) allowed for the use of CGM in the inpatient setting, with many hospitals rapidly implementing CGM with little guidance in a desperate effort to preserve PPE and glucose monitoring abillities.[25,29] Already, this has shown some potential for the intended ancillary benefits, as one report of CGM with remote monitoring in four patients with COVID-19 in Israel showed a 50% decrease in the number of required bedside glucose measurements, as well as a decrease in the risk of staff exposure and care burden.[30] The feasibility of remote real-time CGM use during COVID-19 was also recently reported by Reutrakul and colleagues.[31] In addition, a reduction in hypoglycemia has also been demonstrated using inpatient remote real-time CGM in a recent trial at the Baltimore VA Medical Center.[32] In this study, patients with T2DM on basal-bolus insulin who were considered at-risk for hypoglycemia were randomized to Dexcom G6 CGM with data continuously available to nursing staff in a similar telemetry-like set-up vs. standard inpatient capillary BG checks (approximately four times daily) with a blinded CGM. Among those in the actively monitored CGM group, total per-patient glycemic events <70mg/dL and <54 mg/dL were reduced by 60% (p=0.024) and 89% (p=0.003) respectively compared to standard of care. Reductions were also seen in percent of time spent below <70 mg/dL (0.40% vs. 1.88%, p=0.002) and below <54 mg/dL (0.05% vs. 0.82%, p=0.017). Furthermore, another recent RCT using real-time CGM (G6) showed a modest reduction of time spent in hyperglycemia >250 mg/dL (-11.41%) with a similar approach but including alarms, the TIR (70-180 mg/dl) was 25% for the intervention group and 19.9% in the control group.[33] With a standard protocol using basal-bolus, the TIR (70-180) was ~55% with two different basal insulins.[27]

1.2.2 Hybrid closed-loop insulin therapy

Hybrid closed-loop (HCL) or automated insulin delivery (AID) systems are one of the newest available technologies for diabetes management, designed to make algorithm-based adjustments to insulin infusion using CGM sensor glucose values and trends to maximize time in target glucose range [time in range (TIR), 70-180 mg/dL]. An insulin delivery device (pump) interacts directly with the CGM to adjust insulin continuously according to need in real-time. The use of HCL therapy is being rapidly incorporated into ambulatory DM management and has been shown to be safe and effective in diverse populations and settings.[34-37] The proposed Omnipod5/Horizon AID system, employing a personalized MPC algorithm, is a novel device that has been evaluated for safety in children and adults with T1DM, performing well under supervised free-living conditions.[35] It has also been shown to cope effectively with common glycemic control challenges in the outpatient setting, including daily physical activity and unrestricted meals in adults with T1DM.[35,36] This system has demonstrated remarkable achievement of glycemic targets in children, adolescents, and adults with T1DM,[35] including in those at the highest risk of hypoglycemia.[36] For example, use of the Omnipod Horizon HCL system resulted in a very low risk of hypoglycemia with a median of 0.9% ± 1.3% of glucose readings <70 mg/dL and 0.09% ± 0.25% <54 mg/dL in adults with T1DM treated as

outpatients.[38] They achieved 72.5% TIR (70-180 mg/dL), a mean glucose of 154 ± 15 mg/dL and a coefficient of variation (CV) of 30 ± 6%.[38]

The system provides a low rate of hypoglycemia with <2% of sensor glucose readings <70 mg/dL, while achieving over 70% TIR.[38] The pediatric subjects in the Omnipod 5 pivotal study entered the study with a mean glucose of 185 ± 23 mg/dL with 51 ± 13.3% TIR.[38] Other initial trials of this AID system showed percentage of TIR above 70% in different age groups with various challenges applied to the AID system **(Table 1)**.

Table 1. Initial studies of the Omnipod5/Horizon AID system.

**IDE**	**G160169**	**G160169**	**G170012**	**G170012**	**G170143**	**Omnipod 5**	**Omnipod 5**
	S003	S003	S001	S001	S002	Pivotal – first 4-9 weeks	Pivotal – first 4-9 weeks
**Age group (years)**	Adults	Adolescents	Adults	Adults	Adults	Adults (14-70)	Pediatrics (6-14)
**Challenge**	100% meal bolus	100% meal bolus	Meals	Exercise	Free-living	Free-living	Free-living
**Sample size (n)**	10	12	12	12	11	18	18
**Study duration**	36 hours	36 hours	54 hours	54 hours	96 hours	4-9 weeks	4-9 weeks
**Mean age (yrs)**	41.7±18.1	14.6±1.5	35.4±14.2	36.6±14.4	28.3±7.5	35±11	10.6±1.8
**DM duration (yrs)**	23.7±15.3	4.6±3.3	16.5±9.3	21.6±15.7	13.4±6.0	17±12	5±3
**A1C (%)**	7.4±0.8	8.2±0.9	7.7±0.9	7.6±1.1	7.4±1.1	7.1±0.8	7.8±0.9
**Mean glucose**	155.0±14.8	153.4±21.6	153.4±15.3	136.0±14.3	149.7±11.3	155 ± 15	158 ± 10
**Sensor Glucose (% time)**							
**70-180 mg/dL**	73.0±15.0	72.6±15.5	76.1±8.0	85.1±9.3	73.7±7.5	73.8 ± 9.1	70.1 ± 5.2
**< 50 mg/dL**	0.0 ± 0.1	0.2 ± 0.3	0.0 ± 0.1	0.1 ± 0.2	0.1±0.2	0.1 (0.0, 0.2)	0.2 (0.0, 0.5)
**< 70 mg/dL**	0.7±1.2	2.0±2.4	0.6±0.9	1.4±1.3	1.9±1.3	0.9 (0.5, 1.3)	1.2 (0.9, 2.2)
**> 180 mg/dL**	26.3±14.4	25.4±16.1	23.3±8.5	13.5±9.5	24.5±7.7	25.0 ± 9.1	28.3 ± 5.2
**≥ 250 mg/dL**	3.6±3.7	4.9±6.3	4.5±3.6	1.8±2.4	4.5±4.2	5.6 ± 6.1	8.6 ± 4.1

While some recent efforts have been made to move these new technologies to the inpatient setting to improve hospital diabetes management,[23,24] inpatient data for HCL/AID use is limited. Several studies from the United Kingdom have shown promising results in glycemic control parameters in specific hospitalized populations.[6-8] For example, an inpatient trial by Bally et al. enrolling non-ICU patients with DM showed those assigned to closed-loop therapy achieved 65% TIR compared to 41% with usual care (P<0.001) without an associated increase in hypoglycemia.[6] Two recent studies reporting data on glycemic control using real-time CGM in the inpatient setting showed lower percent TIR achieved in this setting for patients on subcutaneous insulin regimens [median TIR 25.31% (11.78-42.97)[33]; mean TIR 56.6 ± 25.6% and 57.5 ± 25.1%[27]].

The Omnipod5/Horizon AID system has not been tested in hospitalized adults; however, among available options, we believe it is uniquely fashioned toward assimilation and optimal performance in the inpatient setting. The system provides significant hypoglycemia protection, with an automated “hypoglycemia protect” feature which simultaneously raises the algorithm’s glucose target to 150 mg/dL, decreases basal insulin delivery by 50%, and decreases the

aggressiveness of any insulin delivery in response to increasing glucose levels. Glucose rate of change information is also accounted for at the time of boluses, such that a reverse correction is calculated and instituted to prevent hypoglycemia when glucose levels are below the target at the time of a bolus insulin dose. As described previously, in the outpatient setting, these have resulted in extremely low rates of hypoglycemia,[38] and will be both practical and valuable in managing patients with variable insulin requirements. Automated features are supplemented by patient-specific physician input, as providers also have control over the insulin-to-carbohydrate ratios (ICR) and correction/sensitivity factors (ISF) for manual dose calculations.

Another key aspect of this system that makes it particularly attractive for inpatient use lies in its remote operation capability. Most HCL/AID insulin delivery systems require a physical connection between patient and device wherein the insulin pump is attached to the infusion site cannula by variable lengths of plastic tubing. However, the Omnipod is the only commercially available insulin pump that does not employ any tubing to connect the subcutaneous insulin infusion site to the insulin reservoir; both are contained within the disposable "Pod" worn by the patient. Additionally, the Omnipod5/Horizon AID system employs a physically independent controller (PDM smartphone) that is used to monitor CGM values and HCL functional status, perform infusion site (pod) changes, and manually control insulin infusion when required ("open-loop" setting), while also allowing closed-loop/automated insulin delivery to continue in the physical absence of the controller.[35] This could enable HCW to interact with the pump, including delivering insulin and changing delivery settings from *outside* the patient's room if needed. **As such, this device independence offers a unique opportunity for insulin delivery in the hospital to improve glycemic control with remote management capabilities.**

A third key aspect of the Omnipod5/Horizon AID system is that all of the components are intended for single-person use and have relatively low start-up costs compared to other available insulin pumps. The Omnipod5/Horizon AID system (PDM, pod) costs much less than other available closed-loop systems. Pumps used by these other systems can cost on the order of $4,000 to $8,000 and are intended to be used for at least four years before replacement. This makes it possible to use the Omnipod5/Horizon AID system in the hospital without needing to own any component of the system prior to admission, including the insulin pump (PDM). This is especially relevant for patients requiring isolation.

Our overarching ***central hypothesis*** is that with the Omnipod 5/Horizon AID system, AID therapy is feasible and safe in the hospital, achieves superior glycemic control compared to the current standard of care, and reduces the frequency of patient-staff encounters associated with PPE use. This protocol is an initial implementation trial to assess feasibility of and barriers to larger-scale inpatient deployment of this system (***Aim 1***) and to describe early experience with its use and glycemic control in the hospital (***Aim 2***). Results generated from this trial will serve as preliminary data in the creation of a large multi-institution randomized controlled trial (RCT) to assess superiority compared to standard of care insulin therapy, including the potential for future use in those with COVID-19 infection or other conditions requiring isolation precautions.

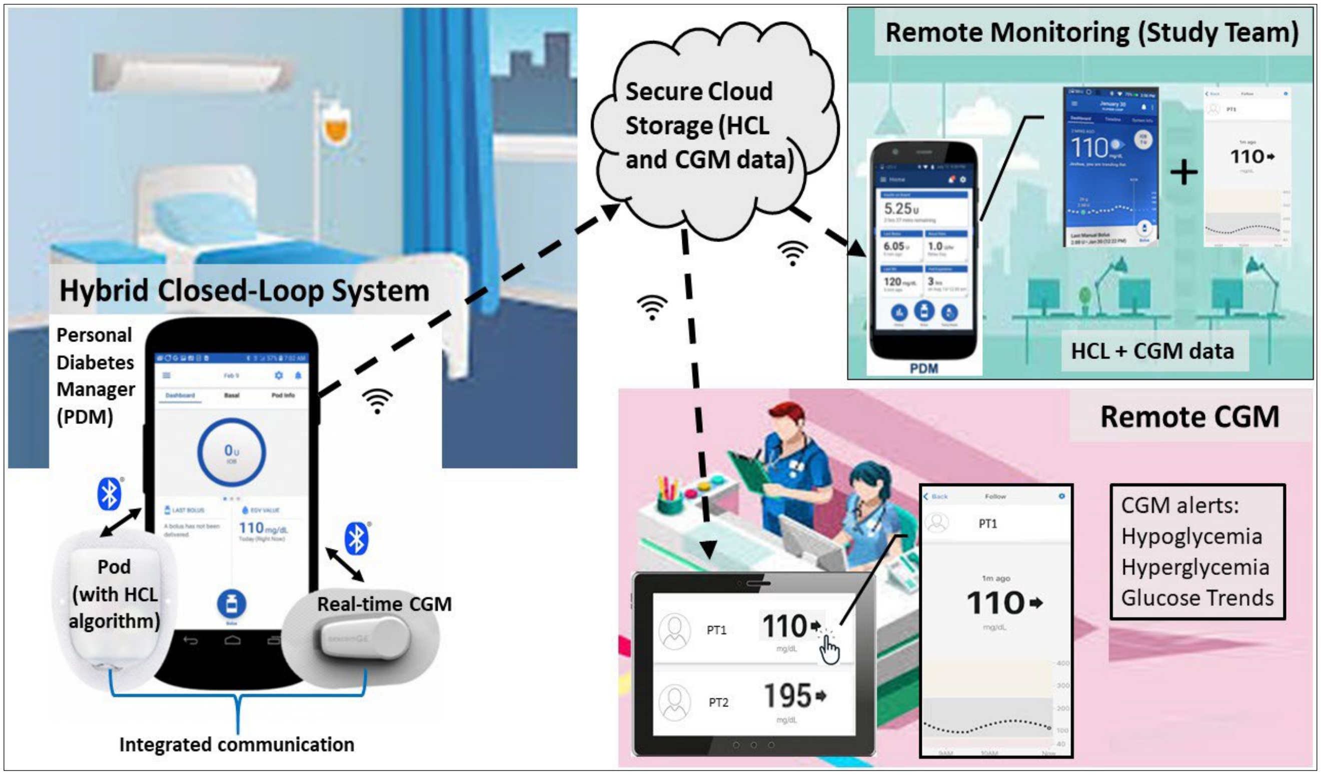

Figure 3. Schematic for Omnipod5/Horizon AID and Dexcom G6 CGM remote monitoring in the hospital.

<u>Aim 1</u>: To test the functional operability and applied use of the Omnipod 5/Horizon AID system in patients with DM admitted to the hospital (Figure 3). We will test deployment of the HCL system in each of the three institutions that plan to participate in the eventual RCT. Eighteen patients with pre-existing insulin-treated DM admitted to a non-ICU medical-surgical floor will receive HCL insulin therapy. Glucose monitoring will transition from intensified POC testing (phase 1, n=9) to standard of care POC testing (phase 2, n=9). To investigate operability (**Aim 1.1**), we will specifically examine the proportion of time spent in HCL settings after initial CGM validation, percentage of time with CGM readings, and percentage of bolus or corrective insulin doses given based on CGM values. We will also assess proportion of CGM values within ±15 and 20% of POC reference values for glucose levels ≥70 mg/dL and ±15 or 20 mg/dL for POC glucose levels <70 mg/dL (%15/15, %20/20). To assess patients' perception of AID in the hospital we will conduct a qualitative assessment with a survey including open-ended feedback questions (**Aim 1.2**).

Hypothesis 1: Inpatient HCL therapy with the Omnipod 5/Horizon AID system functions well/consistently in the hospital and is supported by patients and staff.

Aim 2: To assess glycemic control with the Omnipod 5/Horizon AID system in the inpatient setting. To describe glucometric data associated with inpatient HCL use (**Aim 2.1**), we will analyze and report CGM data including percent time in target glucose range (TIR, 70-180 mg/dL), number of hypoglycemic (<70 mg/dL) and clinically-important hypoglycemic (<54 mg/dL) episodes per patient and per patient-day, percent time below range (TBR, <70mg/dL), percent time in clinically-important hypoglycemia range (<54 mg/dL), percent time above range (TAR, >180 mg/dL), percent time in severe hyperglycemia (>250 mg/dL), coefficient of variation (CV) and standard deviation (SD). To assess HCL initiation settings and the need for setting adjustments in hospitalized patients **(Aim 2.2**), we will evaluate the frequency of adjustments to insulin infusion doses including basal rate, insulin-to-carbohydrate ratio (ICR), and insulin sensitivity factor (ISF) in response to: 1) clinically-important hypoglycemia (<54 mg/dL) and 2) prolonged hyperglycemia (>250 mg/dL for >1 hour).

Hypothesis 2: The Omnipod 5/Horizon AID system is safe and provides adequate (>55% TIR) glycemic control in non-ICU hospitalized patients.

This proposal addresses multiple unique opportunities for the transformation of inpatient diabetes care, including: the **(1)** translation of HCL/automated insulin delivery to the inpatient setting, **(2)** the efficacy of these technologies in improving inpatient glycemic control and rates of iatrogenic hypoglycemia in patients admitted with DM and diverse admission diagnoses, and **(3)** the potential for future studies testing the use of remote HCL and CGM technology for administration of insulin, as it pertains to patients who have isolation precautions, including for COVID-19.

1.3 Preliminary Data

Our preliminary data from a pooled analysis of inpatient CGM studies including 4,067 matched CGM and POC glucose values from non-ICU patients with T1 and T2DM showed an overall mean absolute relative difference (MARD) of 12.8%. The MARD decreased with time as expected (12 hours: 16.4%, 24 hours: 14.4%, All values: 12.8%). The proportion of CGM values with in ±15, 20 and 30% of POC reference values for glucose levels >100 mg/dL and ±15, 20 or 30 mg/dL for POC glucose levels ≤100 mg/dL (%15/15, %20/20, %30/30) increased between the first 12-hours (57.0, 69.2, 85.9%)and 24-hours (63, 75.6, 89.2%) of sensor life. The overall proportion of CGM values meeting %15/15, %20/20, %30/30 criteria were 68.7, 81.7, 93.8%, respectively **(Table 2)**. MARD and median ARD varied according to categories of hemoglobin level and POC glucose level strata with minor variations according to renal function categories. The highest MARD was observed for patients with glucose levels in the hypoglycemic range (50-

	CGM vs Capillary POC (first 12 hours)	CGM vs Capillary POC (first 24 hours)	CGM vs Capillary POC (all data)
Paired readings, n	263	627	4067
MARD, %	16.4	14.4	12.8
Median ARD, % (IQR)	12.5	11.1	10.1
%15/15, %20/20, %30/30	57.0, 69.2, 85.9	63.0, 75.6, 89.2	68.7, 81.7, 93.8

Table 2. CGM accuracy metrics in non-critically ill hospitalized patients with diabetes (preliminary data, unpublished

70mg/dL; MARD 14.5%) and for those with severe anemia (hemoglobin <7g/dL; MARD 17.8%), **Figure 4**.

A Clarke error grid (CEG) analysis of all matched pair data showed 98.7% of values falling in

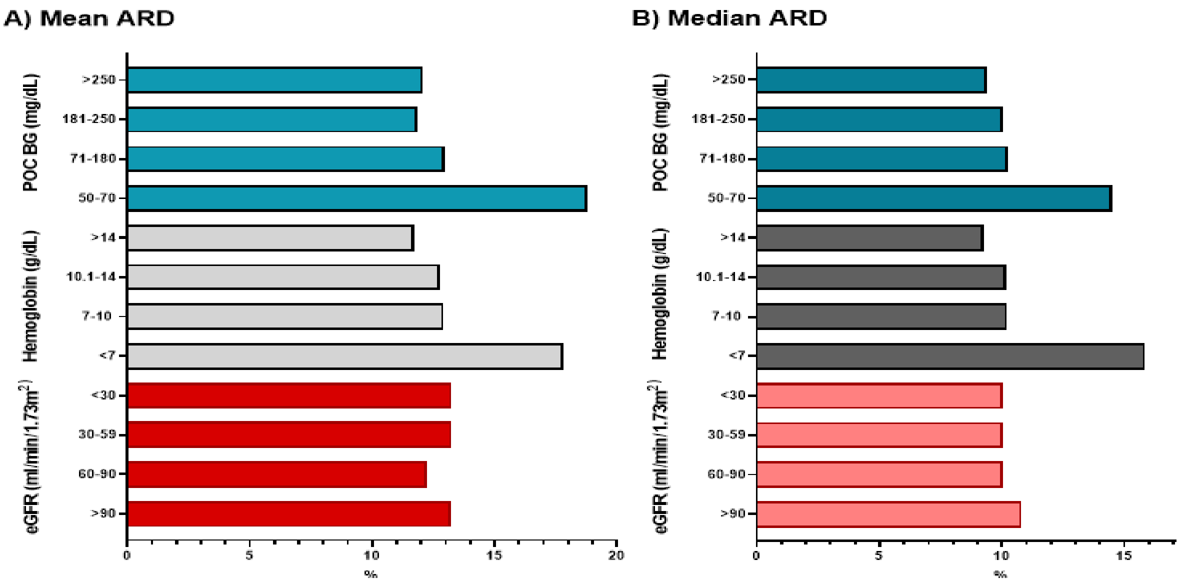

Figure 4. MARD and median ARD comparing CGM with POC in non-ICU hospitalized patients with diabetes, according to eGFR, glucose, and hemoglobin categories (preliminary data, unpublished).

CEG Zones A+B (Zone A, 80.9%; Zone B, 17.8%; Zone C, 0.1%; Zone D, 1.1%, Zone E, 0.0%), **Figure 5.**

At Emory, for patients in the ICU with COVID-19, we have already begun using the Dexcom G6 CGM in conjunction with Glucommander-directed IV insulin therapy. Conceptually, this system parallels closely what we here propose to undertake with HCL therapy. Like HCL, Glucommander uses an algorithm to adjust insulin delivery based upon frequent glucose data input (every 1-2 hours); however, in this case, insulin is provided intravenously, as is appropriate for critically ill patients. As such, beyond the CGM, the components of this system differ considerably from the Omnipod 5/Horizon, which automates

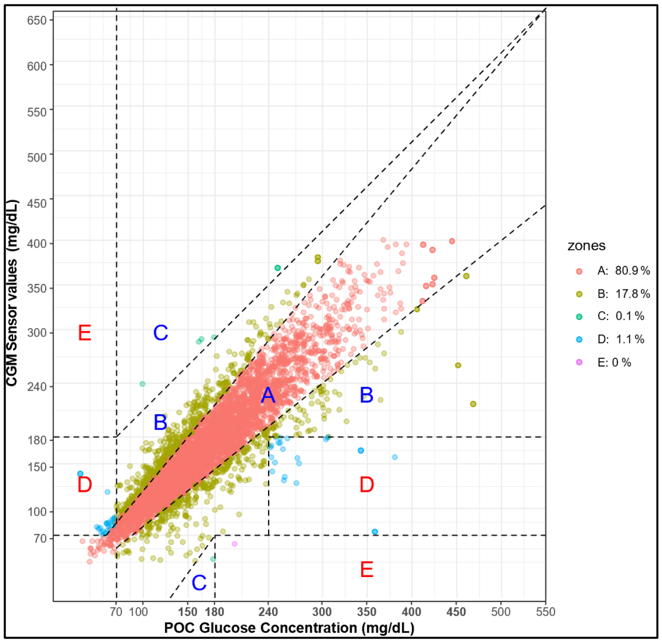

Figure 5. Clark Error Grid analysis of inpatient CGM values compared to POC glucose testing (preliminary data, unpublished).

subcutaneous insulin delivery. In our early experience with the Dexcom G6/Glucommander system, we have observed reliable adjustments to IV insulin infusion based on the CGM values using an intermittent validation protocol comparing CGM to POC glucose values to ensure ongoing CGM accuracy. We have used a similar protocol for routine validation of CGM readings in the ICU as we have proposed in this study. This involved creation of an embedded glucose documentation and validation tool in the electronic health record (EHR) system (EPIC) itself, which after receiving the necessary POC glucose values, advises nursing staff on the necessary accuracy range in which the CGM glucose value must lie in comparison to the POC glucose entered (±20% for glucose values over 100 mg/dL), **Figure 6.** Criteria were also developed to ensure ongoing sensor reliability, with recommendations to revert back to POC glucose testing in cases of: 1) CGM sensor signal loss, 2) loss of CGM trend arrow, and 3) rapid changes in sensor glucose values (>3 mg/dL/min). To investigate feasibility and reliability of the Omnipod5/Horizon AID system in the inpatient setting, we assessed the ability of the pump (pod) and CGM devices to communicate with one another and with the Personal Diabetes Manager (PDM). The PDM is a dedicated android smartphone for the Omnipod5/Horizon and Dexcom applications, which allows for remote control of the insulin pump and reception of CGM glucose values. Reliability of remote bolus insulin delivery and glucose monitoring in COVID-19-specific rooms was tested at Stanford and Emory. The CGM was connected to a “walk-about” sensor simulator and paired with the HCL pod. The CGM and pump (HCL pod) were separated from each other by 3-4 feet to simulate placement on a patient. Because of current PPE shortages, and to avoid unnecessary personnel exposure risk on an active COVID-19 unit, only unoccupied rooms were tested. Communication was tested from the patient inside the room to the PDM stationed outside the room after closing room doors: (1) in different locations within the patient room (including bathroom); (2) with staff personnel acting as an intervening body; (3) in proximity to multiple activated patient care devices with interference potential (telemetry monitor, lights, TV, EKG, ultrasound, iPad, IV pumps); and (4) with variable external PDM locations (wall, hall table).

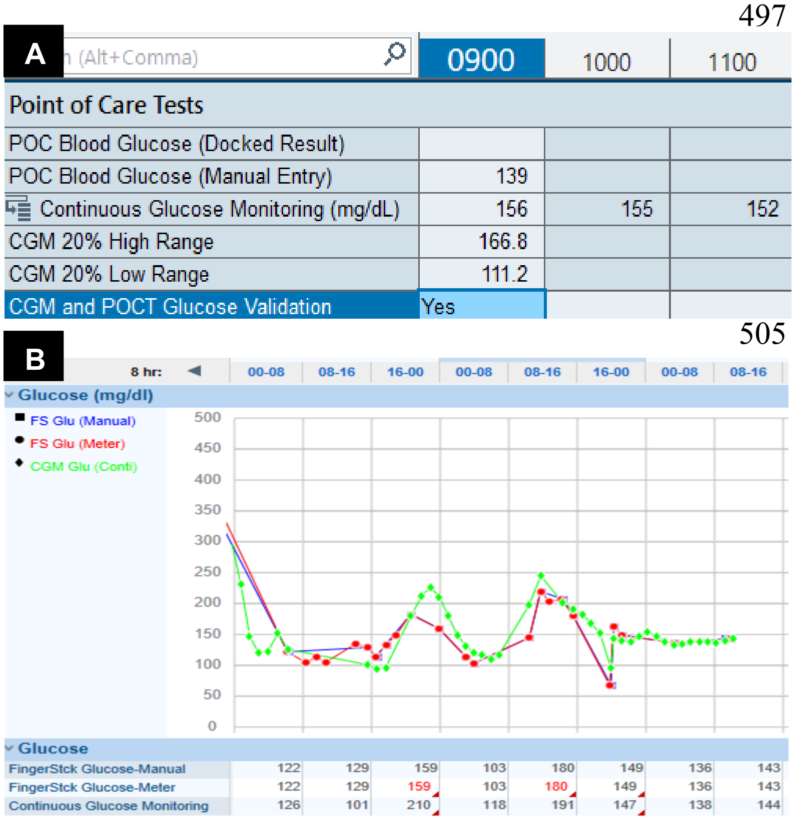

Figure 6. EHR documentation and validation. A) flowsheet shows upper and lower 20% range of POC to document validation (yes/no), B) remote EHR access to documented CGM values trend (green) and POC values.

Throughout the testing, and in every room for each configuration, CGM data remained continuous, with stable readings every five minutes. Each remote bolus was transmitted successfully. An 18-hour overnight test was also completed without any lapse in CGM data or HCL functionality at Stanford.

1.4 Potential Risks and Benefits of the [Investigational Device]

1.4.1 Known Potential Risks

The protocol constitutes greater than minimal risk.

1.4.1.1 Hyperglycemia and Hypoglycemia Risks

The Hybrid Closed Loop System (Omnipod5/Horizon) will have a glucose target lower than what is in the typical range for target glucose in most hospitals. As such, there may be greater risk for hypoglycemia. However, with use of CGM and close monitoring by the study team and hospital staff, this risk should be small. In outpatient studies of patients with T1DM using the Omnipod5/Horizon AID system, the median percent of glucose values <70 mg/dL was <2%. Hypoglycemia, if it occurs, could potentiate a cardiac arrhythmia. If hypoglycemia is severe enough a seizure, loss of consciousness, and even death is possible. These are risks common to all people on insulin therapy.

Hyperglycemia is a known risk with having diabetes, and can occur with rapid changes in diet, or the use of medications such as glucocorticoids, or significant worsening of an underlying medical condition which can result in resistance to the effect of insulin on lowering glucose levels. The pod only uses rapid-acting insulins, so if an insulin infusion catheter was dislodged or became kinked or the insulin flow was blocked, then there is an increased risk of developing hyperglycemia and with hours of interrupted insulin delivery this can lead to ketosis or diabetic ketoacidosis (DKA) which can lead to shock, coma, or death. The glucose values will be monitored continuously and remotely by study staff, so prolonged hyperglycemia is unlikely to occur.

Hypoglycemia and hyperglycemia could also result if there is over or under delivery of insulin due to a device defect, failure or malfunction of any of the components of the system including communication issues, or sensor issues.

Although there is potential for harm from glucose management that is more intensive with a lower glucose goal than standard glucose management, the potential benefit exceeds the potential risk since outcomes of hospitalized diabetic individuals with uncontrolled dysglycemia frequently are poor.

Once the study is completed, participants will need to be transitioned from AID to a subcutaneous insulin regimen. The study team will have knowledge of insulin doses delivered by the Omnipod 5/Horizon AID system, which will be converted into a standard basal-bolus insulin regimen with corrective insulin. This transition to usual care could result in episodes of hypoglycemia or hyperglycemia.

We do not foresee any long-term risks from participating in this study.

The study principal investigators at each site will be closely monitoring for hypoglycemia, hyperglycemia, and device function (errors or failures). The site investigator will be notified when the amount of hypoglycemia exceeding a pre-specified threshold has occurred. Any severe hypoglycemia, DKA, or unanticipated device issues will be reviewed by the Medical Monitor.

1.4.1.2 Other risks

Insertion of the pod infusion set catheter and/or the sensor wire can result in bruising, bleeding, redness, induration, infection, pain and discomfort. The adhesives used to secure the pod and CGM sensor can cause local skin reactions, itching, redness, and allergic reactions to the adhesives.

On rare occasions the CGM sensor wire may break off or be retained under the skin. Rarely this could cause local redness, swelling, or pain at the insertion, and even require surgical removal.

Blood draws as well as fingersticks for blood glucose monitoring are part of routine care, but are also required by this study. Blood draws and fingersticks may cause local pain, bruising and rarely can result in an infection or fainting.

Loss of confidentiality is a potential risk; however, data are handled to minimize this risk. Data downloaded from the CGM and AID device will be collected for the study as measures of diabetes self-management behaviors. Some people may be uncomfortable with the researchers' having such detailed information about their daily diabetes habits or with the questionnaires.

1.4.2 Known Potential Benefits

The use of AID offers the prospect of direct benefit, such as the potential for improved glycemic control. As described above in this protocol, AID therapy is a Food and Drug Administration (FDA) approved therapy for T1DM in people 7-75 years of age with other AID systems. However, its efficacy in treating hospitalized patients with DM or inpatient hyperglycemia is unknown. At this time, it is not the standard of care for hospitalized patients.

AID may benefit patients if improved glucose control is achieved that reduces the incidence of adverse outcomes due to infection or other conditions that lead to the need for hospitalization. The information generated by the study will have generalizable value to adults with insulin needs who are hospitalized for serious illnesses. If this therapy is well accepted by hospital personnel and hospital physicians, it could become the standard of care for patients with DM who are hospitalized and require insulin therapy. The risk to patients is relatively small and the potential benefit is great if improved glucose control reduces the incidence of adverse hospital outcomes.

1.4.3 Risk Assessment

The risks for events such as DKA and severe hypoglycemia are not expected to be greater than they are with use of AID therapy compared with usual care not using a HCL system. Study team and nursing staff will also be using CGM remote monitoring of their glucose values, which may

reduce the risks of severe low blood glucose levels or prolonged high glucose levels. In addition to monitoring, the CGM validation procedures described in section 3.1 mitigate the risk of bolus or corrective insulin dosing based on an inaccurate CGM reading.

1.5 General Considerations

The study is being conducted in compliance with the policies described in the study policies document, with the ethical principles that have their origin in the Declaration of Helsinki, with the protocol described herein, and with the standards of Good Clinical Practice (GCP).

When feasible, data will be directly collected in electronic case report forms (CRF), which will be considered the source data.

The protocol is considered a significant risk device study, due to the fact that the use of the Omnipod5/Horizon AID system is experimental and not approved for use in a hospital setting. The Dexcom G6 sensor is also not approved for use in a hospital setting. Therefore, an investigational device exemption (IDE) from the U.S. Food and Drug Administration (FDA) is required to conduct the study.

Chapter 2: Study Enrollment and Screening

2.1 Participant Recruitment and Enrollment

Enrollment will proceed with the goal of at least 18 patients completing the study. Up to 30 patients may be enrolled to achieve this enrollment goal. Study participants will be recruited from 3 clinical centers in the United States and no clinical centers outside the United States. All eligible participants will be included without regard to gender, race, or ethnicity.

Potential study participants will be identified clinically from the hospital service for people admitted with insulin-treated DM (T1 or T2) requiring inpatient insulin therapy, and from consults sent to the endocrine service for hyperglycemia management. After a potential candidate for enrollment in the study is identified, their primary physician/inpatient team will be contacted to see if they would like to have the patient approached about the study. If so, one of the research staff, coordinator or research physician will explain the study to the patient to see if they are interested in participating.

Participants: Eligible patients meeting inclusion and exclusion criteria as described below will be invited to participate during hospital admission. IRB-approved informed consent will be obtained by study personnel designated at each site (**Figure 1**).

- Three sites will participate in this study (Emory, Stanford, UVA).
- Eighteen patients will be included; up to 30 patients may be enrolled to achieve this enrollment target (accounting for possible early hospital discharge, mortality and study withdrawal).
- Enrollment will proceed stepwise as below:
- Phase 1: Nine patients will receive AID (Omnipod 5/Horizon HCL with Dexcom G6 CGM) combined with intensified POC glucose monitoring (standard of care before meals and at bedtime, adding overnight glucose assessments at approximately 12AM and 3AM, or approximately every 4 hours as clinically indicated).
- Phase 2: Nine patients will receive AID combined with standard-of-care POC glucose monitoring (before meals and bedtime, or approximately every 6 hours as clinically indicated).

2.2 Informed Consent and Authorization Procedures

Potential eligibility may be assessed as part of a routine-care examination. Before completing any procedures or collecting any data that are not part of usual care, written informed consent will be obtained.

For potential study participants the study protocol will be discussed with study staff. The potential study participant will be given the Informed Consent Form to read. Potential study participants will be encouraged to discuss the study with family members and their personal physicians(s) before deciding whether to participate in the study.

As part of the informed consent process, each participant will be asked to sign an authorization for release of personal information. The investigator, or his or her designee, will review the

study-specific information that will be collected and to whom that information will be disclosed. After speaking with the participant, questions will be answered about the details regarding authorization.

A participant is considered enrolled when the informed consent form has been signed.

2.3 Participant Eligibility

2.3.1 Inclusion Criteria

Individuals must meet all of the following inclusion criteria in order to be eligible to participate in the study.

- Any patient ≥18 years of age with insulin-requiring T1 or T2DM admitted to general (non-ICU) medical-surgical hospital service requiring inpatient insulin therapy.

2.3.2 Participant Exclusion Criteria

Individuals meeting any of the following exclusion criteria at baseline will be excluded from study participation.

- Patients admitted to ICU
- Patients anticipated to require less than 48 hours admission
- Evidence of hyperglycemic crises (diabetic ketoacidosis or hyperosmolar hyperglycemic state)
- Severely impaired renal function (eGFR < 30 ml/min/1.73m^2) or clinically significant liver failure
- Severe anemia with hemoglobin <7 g/dL
- Evidence of hemodynamic instability
- Hypoxia (SpO2 <95% on supplemental oxygen)
- Pre-admission or inpatient total-daily insulin dose >100 units daily
- Mental condition rendering the participant unable to consent or answer questionnaires
- Pregnant or breast-feeding at time of enrollment
- Unable or unwilling to use rapid-acting insulin analogs (Humalog, Admelog, Novolog or Apidra) during the study
- Use of hydroxyurea or high dose ascorbic acid (known interference with CGM system)
- COVID -19 infection or Person under investigation (PUI) on isolation precautions

2.4 Screening Procedures

After informed consent has been signed, a potential participant will be evaluated for study eligibility through the elicitation of a medical history, performance of a physical examination by study personnel and local laboratory testing (if needed) to screen for exclusionary medical conditions.

2.5 Data Collection and Testing

Admitting vital signs and height and weight measurements will be recorded. A limited physical exam to assess the participant's skin and suitability for wearing the study devices will be performed by the study investigator or designee (a physician, resident/fellow, nurse practitioner or a physician assistant).

The following procedures will be performed/data collected/eligibility criteria checked and documented:

- Inclusion and exclusion criteria assessed
- Demographics (date of birth, sex, race and ethnicity)
- Contact information (retained at the site and not entered into study database)
- Medical history
- Concomitant medications
- Physical examination to include:
  - Weight, height
  - Vital signs including measurement of blood pressure and pulse
- Blood glucose obtained at time of admission to the hospital if on insulin prior to admission, or blood glucose at the time insulin therapy was started in the hospital
- Record latest O_2 saturation if one has been obtained
- Order HbA1c if one has not been ordered
- Order Creatinine if one has not been ordered
- Urine or serum pregnancy test for all women who have reached menarche and are premenopausal and are not surgically sterile

2.5.1 Daily data recording

Each day the medications given will be recorded in our database, with the time and dose of the medication. The lowest O_2 saturation, Hct, and highest creatinine will be recorded if they were obtained.

The date and time of any laboratory measurements of serum glucose will be recorded into our database

Chapter 3: Study Procedures

3.1 Omnipod 5/Horizon automated insulin delivery system

All participants will receive treatment with the Omnipod Horizon AID system with integrated Dexcom G6 CGM. These devices will communicate with a patient-specific Samsung smartphone (the PDM) secured within the patient room and remotely monitored by nursing station and study team (see **Figure 3**). AID devices will be placed by approved members of the study team, and study physicians will place orders for insulin doses and be responsible for all changes to the device settings. HCL therapy will continue until discharge or for 10 days from enrollment, whichever is shorter (see discontinuation below). Study procedures will be conducted in medical-surgical floors where study team has provided dedicated in-service training. Staff representatives for the nursing teams will be identified and invited to join the study team. They

will assist with device training and use, as well as facilitate communication and provide feedback from nursing staff to the study team. All study team members will receive dedicated training as outlined in Appendix A.

Nursing staff in medical-surgical units will provide standard-of-care insulin therapy delivered using the investigational device for patients enrolled in the study. Dedicated in-service training about the technology and the study protocol will be conducted by the study team for nursing staff (both AM and PM shifts), Appendix B. Once a patient is identified by the study team, the nursing staff on the floor will be notified the patient will be on automated insulin delivery. The nurse taking care of the patient will receive an additional review of study procedures and the devices. A study team member will review training components with staff taking care of patients assigned to AID therapy as outlined in Appendix C. Study investigators will document enrollment of the patient in the EHR and confirm review of nurse training, including providing confirmation of the 24-hour contact information for the study team (Appendix C).

Specific topics that will be reviewed with the nurse taking care of the patient (Appendix C) include:

1. How to give a bolus using the bolus calculator on the Horizon app using the CGM glucose or a meter BG reading if the CGM reading does not meet accuracy criteria.
2. How the home screen appears when the subject is in open or closed-loop
3. How to assess if the CGM is working properly, i.e. showing a glucose reading and trend.

Only approved research staff will initiate the system and replace any components of the system when required during the study (pods or sensors). Research staff will notify the nurse when there is hyperglycemia and the need for a correction dose.

Patients will not interact with the AID system and will not be provided access to real-time CGM glucose data for self-monitoring. Patients would not receive training or learn how to use the AID system for insulin delivery. The AID and CGM devices for control and monitoring will be secured in a locked container inside of the patient room to prevent unauthorized access to the system. Only trained personnel will be provided access. The secured PDM will be placed within approximately 15 feet of the patient to minimize potential for signal loss. Should the PDM not be communicating with the Pod/CGM, the research and nursing teams will receive an alert for signal loss >30 minutes to ensure communication is re-established.

NOTE: communication with the PDM is not necessary to maintain closed-loop functionality, but is necessary for delivering bolus insulin and for remote system monitoring.

Horizon app setup and AID initiation (to be performed by research staff):

- For safety, a unique identifier will be assigned to the patient and entered into the Horizon App.
- Basic set up will be verified including correct date and time. Two-person verification of pump settings for ICR, ISF, and basal rates will be individually customized and

thereafter adjusted for each patient by the study investigators using their clinical judgment. The target glucose will be set to 120 mg/dL initially. **The appropriateness of these settings will be reviewed every 24 hours by the research physicians at each site.**

- Initial basal rates will be determined using either 60% of current total daily insulin dose (TDD) or 50% of weight-based TDD estimate if not on insulin prior to admission.
- Pod application instructions: Fill a pod, clean skin, place pod, then activate pod. Do not enter sensor transmitter ID until the sensor has been activated on the Dexcom app.
- The pump can remain in manual mode. A temporary basal rate may be required if the patient has long acting insulin on-board.
- To determine where the Dexcom sensor will be inserted and where the pod will be inserted. They should be "in line of sight" of each other, and in areas where the patient is unlikely to be laying on them.
- Dexcom G6 sensor will be inserted as per the user manual. The transmitter ID will be recorded and entered into the Dexcom app on the PDM. The sensor will be inserted and activated using the sensor code. The sensor code will be recorded in the sensor log. Share function will be activated and the nursing station remote monitoring tablet will be invited to follow, as well as the approved research team members. Sensor alerts will be set for a threshold of <80 mg/dL, and >250 mg/dL.
- Once the sensor has been activated on the Dexcom app, the transmitter ID can be activated on the Horizon app. Automated insulin delivery will not commence until the Dexcom CGM accuracy is validated (see below).
- The PDM will be secured in a locked container inside of the patient's room, within approximately 15 feet of the patient.

3.1.1 Dexcom CGM validation

- All blood glucose values used to validate the CGM sensor glucose values will result from hospital point-of-care glucometers. The type of glucometer used by the hospital will be documented in the study source documentation.
- CGM validation should *not* be attempted during times of rapid glucose fluctuations (indicated by one or more vertical CGM trend arrows). In this event, assessment will be attempted after stabilization of glucose, indicated by a single horizontal or angled CGM trend arrow.
- **Validation will be performed at the initiation of a new sensor/transmitter session and confirmed if the CGM sensor glucose value satisfies the below criteria:**
  - CGM glucose is within the range of 20% of BG for BG values **≥70 mg/dL** *[Validation range: (BG x 0.8) to (BG x 1.2)]*
  - CGM glucose is within the range of 20 mg/dL of BG for BG values **<70 mg/dL** *[Validation range: (BG – 20) to (BG + 20)]*
- Once validation is confirmed, the patient will be transitioned to HCL/AID mode.
- If validation criteria are not met within the first 12 hours of sensor wear, the CGM will be calibrated with the POC glucose value, and the system will remain in the manual/open-loop setting. Reassessment for CGM validation will occur every 2 to 8 hours until criteria are met, or until an additional 12 to 24 hours has elapsed. If 12 to

24 hours pass following calibration without a successful validation, a new sensor will be placed, and the process will be reattempted.

- If a successful CGM validation is not achieved after 48 hours despite the above measures, the patient will instead be placed on standard insulin therapy under the care of the primary admitting service.

3.1.2 Ongoing glucose monitoring

POC glucose monitoring will be determined based on stepwise enrollment phase as below:

Phase 1: POC glucose monitoring will occur at a minimum of 6x per day (below) as clinically appropriate

- Before meals, at bedtime, approximately 12AM and 3AM or
- Approximately every 4 hours

Phase 2: Standard of care POC glucose monitoring with additional POC tests as clinically appropriate

- Before meals and at bedtime or
- Approximately every 6 hours

CGM values will be validated as above and prior to any meal or correction insulin bolus. The CGM glucose value will be compared to a concurrent POC glucose value to verify device accuracy (validation criteria as above). If validation criteria are not met by the CGM glucose value, the POC glucose value will be used for bolus or correction insulin dose calculation by the HCL/AID system in lieu of the CGM glucose value.

At investigator discretion, or if >3 consecutive CGM values do not meet validation criteria during ongoing POC glucose monitoring after initial validation, calibration or sensor change will be performed.

Insulin boluses will be given remotely by nursing staff or study team, but doses will be calculated by the HCL/AID system based on current CGM or POC glucose information and carbohydrate content of meal or snack. Correction boluses will also be delivered as advised by the HCL/AID system.

POC glucose assessments will be used instead of CGM sensor values in the setting of: 1) CGM signal loss for >1 hour, 2) loss of CGM trend arrow for >1 hour, and 3) any concerning clinical status changes. The pump will be transitioned to manual/open-loop mode until the issue is corrected or the patient's clinical status is deemed appropriate by study investigators for transition back to HCL/AID mode.

3.1.3 Insulin Delivery

Nursing Training: Nursing staff and hospital staff working with these patients will receive training on how the AID system works, and how to administer insulin boluses, and how to

change an OmniPod5 pod (see competency checklist for health care professionals for using the AID system in appendix A).

- *Basal*: Basal insulin delivery settings will be initiated and adjusted at the discretion of the study investigators. Basal infusion rates will be dictated by the HCL algorithm when in automated (closed-loop) mode.
- *Prandial*: Nursing staff will bolus prandial insulin calculated by the HCL/AID system from current validated CGM (or POC) glucose value and meal size/carbohydrate content using the pre-set ICR.
- *Corrective:* Non-mealtime correctional boluses will be calculated by the HCL system (or determined by the pre-set ISF in open-loop settings), and will be given under the guidance of the research team.
  - NOTE: No CGM-based insulin dosing calculations will be made or administered if CGM validation is not completed and active.
- *Target Glucose:* The target glucose will initially be set at 120 mg/dL. It is adjustable to targets between 110 to 150 mg/dL, and it can be programmed to have higher or lower targets at different times of the day. The research staff will use their clinical judgement to modify the glucose targets for patient safety or to improve glycemic control.
- HCL/Automated Mode: in this mode, validated CGM values are used by the device algorithm to automatically adjust insulin infusion rates to target glucose value of 120 mg/dL.
- Open-loop/Manual Mode: in this mode, insulin delivery depends only on preset basal rates, ICR, ISF and DIA; no CGM glucose data is required, but may be utilized for insulin dose decisions.

3.2 Unit-based monitoring

CGM data will be available to nursing staff at the nursing station on a dedicated tablet screen (or smartphone) and can also be viewed on the PDM stationed inside the patient room in a locked container.

- Nursing staff working with these patients will receiving training on using CGM data and will complete a competency checklist (Appendix A).
- CGM alerts will be programmed at the nursing station (tablet) and on the PDM to alarm for glucose values <80 mg/dL. PDMs will be mobile, as they will need to move with the patient for any transfers.
- For hypoglycemia alarms (CGM glucose <80 mg/dL), nursing staff will assess the patient and perform POC BG. If concurrent POC BG is <80 mg/dL, patient will be treated by institutional hypoglycemia protocol. If concurrent POC BG is >100 mg/dL, POC BG will be reassessed in 1 hour. If CGM glucose remains in hypoglycemic range, but repeat POC BG remains discordant (>80 mg/dL), the study team will be contacted for further recommendations.
- Battery on PDM will be assessed and charged at least daily.

3.3 Remote monitoring by research staff

- The Dexcom Share app for research staff will be set to alarm for hypoglycemia as above, as well as for CGM glucose values >250 mg/dL for >1 hour. The research staff will review patient glucose values, insulin delivery and patient status and determine if a corrective dose of insulin is indicated.
- For unexplained hyperglycemic range CGM values (>300 mg/dL) exceeding 60 minutes, POC glucose values will be obtained hourly for those with T1DM and every 2 hours for those with T2DM to minimize the risk of hyperglycemic crises. If the CGM glucose remains above 300 mg/dL [>1 hour in T1DM or > 2 hours in T2D] following a corrective insulin dose by the AID system, the study team will be alerted and additional correction action will be taken, with the nursing staff assessing the infusion site, changing the pod or initiating alternative insulin delivery methods (i.e. subcutaneous injections or IV insulin infusion).
- Ketones will be assessed at the discretion of the study physician or primary hospital service for any concern about possible ketosis or DKA by drawing a serum sample (beta-hydroxybutyrate) to be processed at the hospital laboratory.
- Study team will be alerted if there are no CGM readings for 30 minutes and will contact the floor teams to assess the situation and reestablish communication.

3.4 Interruption of HCL Therapy

Imaging/Procedures: if pump and CGM must be removed for any incompatible imaging/procedure (e.g. CT, MRI, surgery, electrocautery), management will depend on length of required discontinuation:[39-41]

- *Short: <1 hour*
  BG 180-300: administer corrective bolus insulin
  If BG is >300: consider transition to IV insulin infusion or continued subcutaneous correction protocol
- *Intermediate: 1-3 hours*
  BG 110-180: administer bolus insulin equal to 70% of the sum of basal rate over the prior 2-3 hours (do not administer if BG is <110).
  BG 180-300: administer corrective bolus insulin
  If BG is >300: consider transition to IV insulin infusion or continued subcutaneous correction protocol
- *Long: >3 hours*
  Consider transition to IV insulin infusion or continued subcutaneous insulin regimen within 1 hour of pump discontinuation and determine ability to transition back to HCL post-procedure.
  Surgical intervention: patients will discontinue HCL insulin therapy and will be treated with subcutaneous or IV insulin as per hospital perioperative glycemic management protocol.

ICU transfers: patients requiring ICU transfer for any reason will be managed by ICU team for diabetes management (i.e. subcutaneous insulin or continuous intravenous insulin infusion). HCL/AID will be discontinued, but use of the Dexcom CGM for glucose

monitoring may be continued at ICU team discretion. Upon transfer out of the ICU, patients may resume HCL therapy for diabetes management when appropriate. Patients with worsening clinical condition (hypoxia or hemodynamically unstable) who do not meet ICU transfer criteria may resume the AID system once those conditions are under control.

Emergency use of insulin for non-glucose-related indications: in the event that a patient requires insulin for a non-glucose-related indication, such as an IV insulin dose for the treatment of hyperkalemia, HCL therapy should may be suspended temporarily at the discretion of the investigator until confirmed resolution of the underlying condition (e.g. hyperkalemia resolves). If HCL therapy is suspended for >6 hours, the patient should be transitioned to and maintained on subcutaneous insulin until the underlying condition resolves and HCL therapy can be resumed.

3.5 Discontinuation of HCL/AID

Criteria for discontinuation of HCL therapy for an individual participant, as well as study hold parameters are detailed below in sections *6.6.1* and *6.6.2*.

Chapter 4: Study Devices

4.1 Description of the Investigational Device

The Omnipod5/Horizon™ System is composed of three primary components (**Figure 7**):

- Omnipod5/Horizon™ Controller – Horizon App (PDM) and Algorithm
- Omnipod5/Horizon™ Alternate Controller Enabled (ACE) Pump – Pod
- Dexcom G6 – CGM

The Omnipod5/Horizon™ System will provide automated insulin delivery when connected to a Dexcom G6 CGM. The system is expected to reduce hypoglycemia without incurring an unacceptable increase in hyperglycemia and mean glucose. The system is also expected to reduce the extent and magnitude of hyperglycemia associated with meals. Optimal post-prandial control requires the user to deliver meal boluses as in current "open-loop" therapy, but the normal operation of the control algorithm will be expected to compensate for mismatched meal boluses and prevent prolonged hyperglycemia. The system uses a control-to-target strategy that attempts to achieve and maintain a set target glucose level, with target setpoints at 110 mg/dL, 120 mg/dL, 130 mg/dL, 140 mg/dL and 150 mg/dL. Target setpoints can be adjusted by the user/healthcare provider, and patterns of higher or lower setpoints at different times of the day can be programmed into the Horizon application.

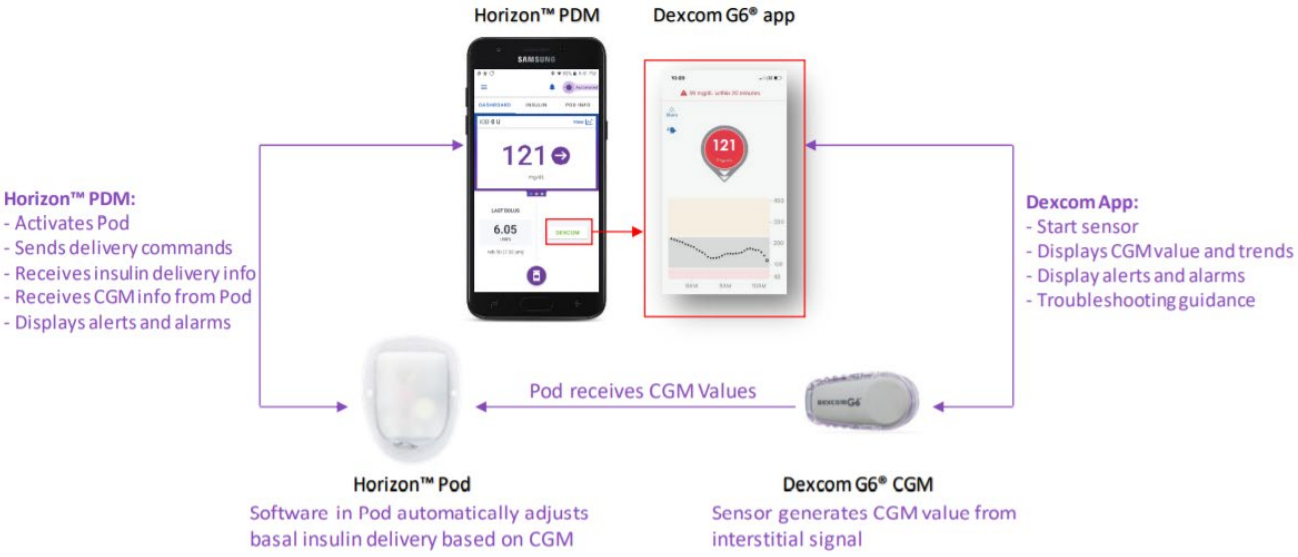

Figure 7. Components of the Omnipod5/Horizon AID and Dexcom G6 CGM system.

The Omnipod5/Horizon™ Controller is composed of two parts: the Horizon application ("app") and the model predictive control (MPC) algorithm on the Pod. The MPC algorithm provides insulin micro-boluses once every 5 minutes based upon the predicted glucose over a 60-minute prediction horizon. Optimal post-prandial control will require meal boluses in the same manner as current pump therapy, but normal operation of the MPC algorithm will compensate for late or missed or underestimated meal boluses and mitigate prolonged hyperglycemia. The MPC algorithm uses the control-to-target strategy to achieve and maintain a set target glucose value,

thereby reducing the duration of prolonged hyperglycemia and hypoglycemia. The MPC algorithm resides on the Pod (Pump) component of the Omnipod5/Horizon™ System (similar to the DASH alternate controller enabled (ACE) pump cleared in K191679, as described further below).

The Horizon™ app will be the primary interface and will be used to start and stop a Pod, program basal and bolus calculator settings for manual mode as well as program settings specific for Automated Mode (hybrid closed-loop).

Manual ("Open-loop") Mode:

In Manual Mode, the Omnipod5/Horizon™ System will function equivalently to the Omnipod® DASH System, which was first cleared under K180045, most recently under K191679. This includes delivering insulin at programmed basal rates and bolus amounts with the option to set temporary basal profiles. The Omnipod5/Horizon™ Controller will also have the ability to function as a sensor augmented pump in Manual Mode, using sensor glucose data provided by the CGM to populate the bolus calculator.

Automated ("Hybrid closed-loop") Mode:

In Automated Mode, the system will support the use of multiple target glucose values, currently intended to be 110-150 mg/dL in 10 mg/dL increments. The research team will program basal rates, glucose targets and bolus calculator settings (see section 3.2). These in turn will inform the MPC algorithm for insulin dosing parameters. The insulin dosing parameters will be adapted over time based on the total daily insulin (TDI) delivered during each Pod use. A temporary hypoglycemia protection mode (Hypo Protect) may be implemented by the user for various time durations during Automated Mode. With Hypo Protect, the algorithm reduces insulin delivery and is intended for use over temporary durations when insulin sensitivity is expected to be higher, such as with discontinuation of steroids, or a significant improvement in illness-related stress, and exercise for ambulatory patients. This function would be enabled at the discretion of the research physicians.

The Omnipod5/Horizon™ System will include two apps on a locked-down smartphone (the Samsung J3), referred to as Personal Diabetes Manager (PDM): the Horizon App and the Dexcom App. This phone has shown good communication between the Pod and Dexcom G6 sensor in the hospital setting, communicating from the hallway to far corners of the patient's room (including bathrooms), with multiple hospital monitoring devices and pumps functioning in the room. The Horizon App, which will have a similar interface to the cleared Omnipod® DASH System (K191679), will allow the use of large text, graphics, and on-screen instructions to prompt the user through set-up processes. It will also be used to program the user's custom basal insulin delivery profile, check the Pod status, initiate bolus doses of insulin, make changes to a patient's insulin delivery profile, handle system alerts and alarms, and enter Automated Mode.

The Dexcom App interface is identical to the current app of the interoperable Dexcom G6 Continuous Glucose Monitoring System (K191450) and will provide CGM data, alerts, and alarms to the user.

The Horizon App and Dexcom App will not directly communicate with one another. Instead, the CGM transmitter will communicate estimated glucose values (EGV) directly to the Pod. The Dexcom transmitter number must be entered into the Horizon App, and this information is sent to the Pod to allow transmission of EGV. The Pod will pair directly to the transmitter to receive EGV for the algorithm and also sends the EGV back to the Horizon App as shown in **Figure 7**.

The Omnipod5/Horizon™ Controller provides the ability to calculate a suggested bolus dose through the use of the bolus calculator. The bolus calculator will have the option to use the EGV, which is communicated to the app via the Pod. If the sensor has passed validation criteria for the day, it is preferred that the EGV be used for correction and meal boluses, since the algorithm will also use the sensor rate of change information in calculating the insulin dose. This allows for less insulin delivery when the estimated glucose is decreasing and more insulin delivery when the estimated glucose is increasing. Insulin-on-board (IOB) is calculated by the algorithm taking into account any manual bolus and insulin delivered by the algorithm.

As with the cleared Omnipod® DASH System, Insulet will utilize a proprietary encrypted security stack embedded within the Bluetooth Low Energy (BLE) communication.

4.1.1 Omnipod5/Horizon™ automated insulin delivery system

The Pod component of the Omnipod5/Horizon™ System is similar to the Omnipod® DASH ACE Pump cleared under K191679. Compared to the DASH ACE Pump (K191679), the Horizon™ ACE Pump (Pod) has additional software to optimize communication to accept inputs from the CGM (Dexcom G6) and the Horizon™ Controller. The insulin delivery mechanism and the patient and fluid contacting components are identical to the DASH Pod.

The Pod is a lightweight, self-adhesive device that the user fills with U-100 rapid-acting insulin and wears directly on their body. The Pod delivers insulin into the user's body through a small flexible tube, called a cannula, based on the commands from the compatible controller. In the Omnipod5/Horizon™ System, the Pod will house the MPC algorithm and communicate directly with the CGM and the Horizon App. The algorithm commands the Pod's insulin delivery in the form of micro-boluses based on predicted glucose values. As with the cleared Omnipod® DASH System, the Pod of the Omnipod5/Horizon™ System will come pre-packaged in a sterile container with a fill needle and a fill syringe. **Figure 8** below is a representation of the Pod. The Omnipod5 has not been approved for commercial sale and has not been tested for use on hospitalized patients and is therefore considered experimental.

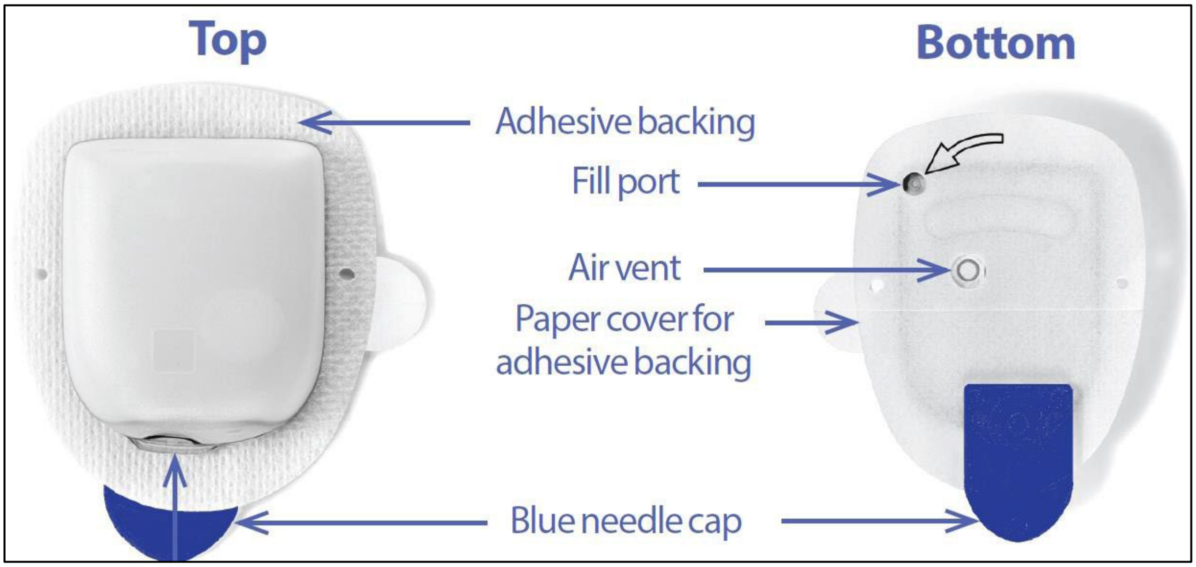

Figure 8: The Pod of the Omnipod5/Horizon™ ACE pump

4.1.2 Continuous Glucose Monitoring

The second component of the Omnipod5/Horizon™ System is the CGM. The Omnipod5/Horizon™ System will be interoperable with a compatible CGM, currently the Dexcom G6 CGM System (K191450). The Pod will communicate with the Dexcom G6 via Bluetooth Low Energy (BLE). Glucose values from the Dexcom transmitter will be sent to the MPC algorithm residing on the Pod and will be used in insulin dosing adjustments. The glucose values from the Dexcom transmitter will be sent independently to the Dexcom App on the controller. The Dexcom G6 sensor is not approved for use in a hospital setting and is therefore considered experimental. Alarms will be set as described in sections *3.1.3* and *3.1.4*.

4.1.3 Horizon Data Portal

Data are securely uploaded from the PDM to the Insulet Cloud by cellular connection. Data are then transferred from Insulet Cloud to the Horizon Data Portal (HDP), which is a platform for data review and management. The HDP runs on an Amazon-based web server. The HDP will provide insights including but not limited to: insulin delivery, time in range, time at each target BG, automated/manual mode comparisons, and time spent in each mode.

The research investigators will have access to all uploaded data and be able to view historical trends.

The Insulet Artificial Pancreas Remote Monitoring System (APRMS) allows remote monitoring of glucose levels and insulin delivery in real-time, allowing remote evaluation of hospitalized patients by research staff.

4.1.4 Blood Glucose Meter and Strips

The point of care glucose meter approved by the hospital will be used for POC blood glucose readings. The meter undergoes quality control testing and the nurses are trained on its use. The specific meter used will vary by hospital.

4.1.5 Study Device Accountability Procedures

Device accountability procedures will be detailed in the site procedures manual.

4.2 Safety Measures

4.2.1 CGM verification of validation

Each new study CGM will be validated prior to use of AID. See section *3.1.1*.

4.2.2 Pump Failure

In the event of an early pod failure, the research staff or nursing staff trained on pod filling and activation will initialize a new pod and remove the old pod. Pods will be disposed of in a biohazard waste container.

4.2.3 Hypoglycemia Threshold Alarm and Safety Protocol

The low glucose threshold alarm setting will initially be set to 80 mg/dL, this may be adjusted by the research investigators as the study progresses, or for an individual patient, but will never be less than 70 mg/dL. See sections *3.1.3* and *3.1.4*.

4.2.4 Hyperglycemia Threshold Alarm and Safety Protocol

During the course of the study and for individual patients, researchers will be permitted to change this setting within a range of 200 to 300 mg/dL. Initially this alarm will be set to 250 mg/dL. See sections *3.1.3* and *3.1.4*.

During the time period when the closed-loop system is operational and active, if a participant's CGM reading is >300 mg/dL for over 1 hour or ≥400 mg/dL at any point, the following steps will be taken: Ketones will be assessed at the discretion of the study physician or primary hospital service for any concern about possible ketosis or DKA by drawing a serum sample (beta-hydroxybutyrate) to be processed at the hospital laboratory.

Chapter 5: Testing Procedures and Questionnaires

5.1 Laboratory Testing

HbA1c: to be ordered after enrollment if not obtained within one month prior to admission.

Urine or serum pregnancy test: to be ordered after enrollment for all female enrollees of childbearing potential.

Additional biologic samples will not be collected or ordered expressly for the purpose of this study. However, certain lab results (i.e. CBC, CRP, D-dimer, or IL-6 levels) obtained during the hospital admission as part of the participant's medical care will be recorded in our data collection.

5.2 Surveys and Questionnaires

We will obtain feedback from patients treated with the Omnipod5/Horizon HCL system.

We acknowledge that the inpatient providers will be the primary interactive users with these devices and also that hospitalized patients may have varying degrees of illness, and therefore limited ability to participate in questionnaires. However, we believe patient input and feedback is also of great potential benefit. As such, we have included patient surveys, but have limited them to 8-items, to be answered if able.

After patients are taken off the system they will be asked to:

- Fill out a brief, 8-item questionnaire (Appendix Z)

Chapter 6: Unanticipated Problem, Adverse Event, and Device Issue Reporting

6.1 Unanticipated Problems

Site investigators will promptly report to the study Principal Investigator, Institutional Review Board (IRB), and to the Medical Monitor all unanticipated problems meeting the criteria below within seven (7) calendar days. For this protocol, an unanticipated problem is an incident, experience, or outcome that meets all of the following criteria:

- Unexpected (in terms of nature, severity, or frequency) given (a) the research procedures that are described in the protocol related documents, such as the IRB-approved research protocol and informed consent document; and (b) the characteristics of the subject population being studied.
- Related or possibly related to participation in the research (possibly related means there is a reasonable possibility that the incident, experience, or outcome may have been caused by the procedures involved in the research)
- Suggests that the research places participants or others at a greater risk of harm than was previously known or recognized (including physical, psychological, economic, or social harm)

6.2 Adverse Events

6.2.1 Definitions

Adverse Event (AE): Any untoward medical occurrence in a study participant, irrespective of the relationship between the adverse event and the device(s) under investigation.

Serious Adverse Event (SAE): Any untoward medical occurrence that:

- Results in death.
- Is life-threatening; (a non-life-threatening event which, had it been more severe, might have become life-threatening, is not necessarily considered a serious adverse event).
- Requires prolongation of current hospitalization.
- Results in persistent or significant disability/incapacity or substantial disruption of the ability to conduct normal life functions.
- Is considered a significant medical event by the investigator based on medical judgment (e.g., may jeopardize the participant or may require medical/surgical intervention to prevent one of the outcomes listed above).

Unanticipated Adverse Device Effect (UADE): Any serious adverse effect on health or safety or any life-threatening problem or death caused by, or associated with, a device, if that effect, problem, or death was not previously identified in nature, severity, or degree of incidence in the investigational plan or application (including a supplementary plan or application), or any other

unanticipated serious problem associated with a device that relates to the rights, safety, or welfare of participants (21 CFR 812.3(s)).

Adverse Device Effect (ADE): Any untoward medical occurrence in a study participant which the device may have caused or to which the device may have contributed (Note that an Adverse Event Form is to be completed in addition to a Device Deficiency or Issue Form, unless excluded from reporting as defined in section *6.2*). *An event that occurs solely due to user error in which the device functions properly generally will not be considered an ADE unless it is determined that the instructions on the screen of the device or user manual (or similar training materials) may have contributed to the event (note: the event may still meet criteria for reporting as an adverse event).*

Device Complaints and Malfunctions: A device complication or complaint is something that happens to a device or related to device performance, whereas an adverse event happens to a participant. A device complaint may occur independently from an AE, or along with an AE. An AE may occur without a device complaint or there may be an AE related to a device complaint. A device malfunction is any failure of a device to meet its performance specifications or otherwise perform as intended. Performance specifications include all claims made in the labeling for the device. The intended performance of a device refers to the intended use for which the device is labeled or marketed (21 CFR 803.3). Note: for reporting purposes, sites will not be asked to distinguish between device complaints and malfunctions.

6.2.2 Reportable Adverse Events

For this protocol, a reportable adverse event includes any untoward medical occurrence that meets one of the following criteria:

1. An ADE as defined in section *6.2.1*, unless excluded from reporting in section 8.3
2. An AE as defined in section *6.2.1* occurring in association with a study procedure
3. An AE as defined in section *6.2.1* that affects the participant’s ability to complete any study procedures
4. Hypoglycemia meeting the definition of severe hypoglycemia as defined below
5. Diabetic ketoacidosis (DKA) as defined below or in the absence of DKA, hyperglycemia or ketosis event meeting the criteria defined below.

Hypoglycemia and hyperglycemia not meeting the criteria below will not be recorded as adverse events unless associated with an Adverse Device Effect. Skin reactions from sensor placement are only reportable if severe and/or required treatment.

All reportable AEs—whether volunteered by the participant, discovered by study personnel during questioning, or detected through physical examination, laboratory test, or other means—will be reported on an AE form. Each AE form will be reviewed by the Medical Monitor to assess for safety and study continuation.

Refer to the JCHR IRB Investigator Handbook for detailed information regarding safety event reporting to the IRB.

6.2.3 Hypoglycemic Events

Hypoglycemia not associated with an Adverse Device Effect is only reportable as an Adverse Event when either of the following parameters are met:

- The episode is associated with acute severe cognitive impairment, including incoherence, disorientation, and/or combativeness, seizure, or loss of consciousness. Note: If plasma glucose measurements are not available during such an event, neurological recovery attributable to the restoration of plasma glucose to normal is considered sufficient evidence that the event was induced by a low plasma glucose concentration.
- Capillary glucose is <40 mg/dL

When a hypoglycemic event meets the above reporting requirements, a Hypoglycemia Form should be completed in addition to the Adverse Event Form. Severe hypoglycemia events should be considered to be serious adverse events with respect to reporting requirements.

6.2.4 Hyperglycemic/Ketotic Events

Hyperglycemia not associated with an Adverse Device Effect is only reportable as an Adverse Event if the event involved DKA. Hyperglycemic events are classified as DKA if the following are present:

- Symptoms such as polyuria, polydipsia, nausea, or vomiting;
- Serum ketones >1.5 mmol/L or large/moderate urine ketones;
- Either arterial blood pH <7.30, venous pH <7.24, or serum bicarbonate (or CO_2) <15 mEq/L
- DKA is suspected as either the primary or a contributing cause for these findings

When a hyperglycemia/ketotic event meets the above reporting requirements, an Adverse Event Form should be completed. Events meeting DKA criteria should be considered serious adverse events with respect to reporting requirements. Hyperglycemia events not meeting criteria for DKA generally will not be considered as serious adverse events unless one of the SAE criteria in section *6.2.1* is met.

6.2.5 Relationship of Adverse Event to Study Investigational Device

The study investigator will assess the relationship of any adverse event to be related or unrelated by determining if there is a reasonable possibility that the adverse event may have been caused by the study device.

To ensure consistency of adverse event causality assessments, investigators should apply the following general guideline when determining whether an adverse event is related:

- ***Unrelated*:** The AE is clearly not related to a study drug/device and a likely alternative etiology exists such as an underlying disease, environmental or toxic factors or other therapy.
- ***Unlikely Related*:** The AE does not follow a reasonable temporal sequence during or after use of study drug/device and a more likely alternative etiology exists such as an underlying disease, environmental or toxic factors, or other therapy.
- ***Possibly Related*:** The AE occurred in a reasonable time during or after use of study drug/device; but could be related to another factor such as an underlying disease, environmental or toxic factors, or other therapy; and there is a possible, though weak, scientific basis for establishing a causal association between the AE and the study drug/device.
- ***Probably Related*:** The AE occurred in a reasonable time during or after use of study drug/device; is unlikely to be related to another factor such as an underlying disease, environmental or toxic factors, or other therapy; and there is a plausible, though not strong, scientific basis for establishing a causal association between the AE and the study drug/device.
- ***Definitely Related*:** The AE occurred in a reasonable time during or after use of study drug/device; cannot be explained by another factor such as an underlying disease, environmental or toxic factors, or therapy; and there is a strong scientific basis for establishing a causal association between the AE and the study drug/device.
- ***Not Assessable*:** Causality of an adverse event cannot be judged because information is insufficient or contradictory, and which cannot be supplemented or verified.

6.2.6 Severity (Intensity) of Adverse Events

The severity (intensity) of an adverse event will be rated on a three-point scale: (1) mild, (2) moderate, or (3) severe. A severity assessment is a clinical determination of the intensity of an event. Thus, a severe adverse event is not necessarily serious. For example, itching for several days may be rated as severe, but may not be clinically serious.

- MILD: Usually transient, requires no special treatment, and does not interfere with the participant’s daily activities.
- MODERATE: Usually causes a low level of inconvenience, discomfort or concern to the participant and may interfere with daily activities but is usually ameliorated by simple therapeutic measures and participant is able to continue in study.
- SEVERE: Interrupts a participant’s usual daily activities, causes severe discomfort, may cause discontinuation of study device, and generally requires systemic drug therapy or other treatment.

6.2.7 Expectedness

For a serious adverse event that is considered possibly related to study device, the Medical Monitor will classify the event as unexpected if the nature, severity, or frequency of the event is not consistent with the risk information previously described in section *1.4*

6.2.8 Coding of Adverse Events

The Medical Monitor will review the investigator's assessment of causality and may agree or disagree. Both the investigator's and Medical Monitor's assessments will be recorded. The Medical Monitor will have the final say in determining the causality as well as whether an event is classified as a serious adverse event and/or an unanticipated adverse device effect.

6.2.9 Outcome of Adverse Events

The outcome of each reportable adverse event will be classified by the investigator as follows:

- RECOVERED/RESOLVED – The participant recovered from the AE/SAE without sequelae. Record the AE/SAE stop date.
- RECOVERED/RESOLVED WITH SEQUELAE – The event persisted and had stabilized without change in the event anticipated. Record the AE/SAE stop date.
- FATAL – A fatal outcome is defined as the SAE that resulted in death. Only the event that was the cause of death should be reported as fatal. AEs/SAEs that were ongoing at the time of death; however, were not the cause of death, will be recorded as "resolved" at the time of death.
- NOT RECOVERED/NOT RESOLVED (ONGOING) – An ongoing AE/SAE is defined as the event was ongoing with an undetermined outcome.
  An ongoing outcome will require follow-up by the site in order to determine the final outcome of the AE/SAE.
  The outcome of an ongoing event at the time of death that was not the cause of death, will be updated and recorded as "resolved" with the date of death recorded as the stop date.
- UNKNOWN – An unknown outcome is defined as an inability to access the participant or the participant's records to determine the outcome (for example, a participant that was lost to follow-up).

If any reported adverse events are ongoing when a participant completes the study (or withdraws), adverse events classified UADEs will be followed until they are either resolved, or have no prospect of improvement or change, even after the participant has completed all applicable study visits/contacts. For all other adverse events, data collection will end at the time the participant completes the study. Note: participants should continue to receive appropriate medical care for an adverse event after their participation in the study ends.

6.3 Reportable Device Issues

All UADEs and ADEs as defined in section *6.2.1* will be reported on both a device issue form and AE form, except for skin reactions from CGM sensor placement or pump infusion set placement that do not require pharmacologic treatment. As noted in section *6.2.1*, events that occur due to user error generally will not require completion of a device issue form.

Device complaints and device malfunctions will be reported except in the following circumstances. These occurrences are expected and will not be reported on a Device Issue Form assuming criteria for a UADE or ADE have not been met:

- CGM sensor lasting fewer days than expected per manufacturer
- CGM tape adherence issues
- Battery lifespan deficiency due to inadequate charging or extensive wireless communication
- Intermittent device component disconnections/communication failures not requiring system replacement or workaround/resolution not specified in user guide/manual.
- Device issues clearly addressed in the user guide manual that do not require additional troubleshooting

6.4 Timing of Event Reporting

SAEs possibly related to a study device or study participation and UADEs must be reported to the Coordinating Site (Emory University) within 24 hours of the site becoming aware of the event. This can occur via phone or email, or by completion of the online serious adverse event form and device issue form if applicable. If the form is not initially completed, it should be competed as soon as possible after there is sufficient information to evaluate the event. All other reportable ADEs and other reportable AEs should be submitted by completion on the on-line form within 7 days of the site becoming aware of the event.

The Coordinating Site will notify all participating investigators of any adverse event that is serious, related, and unexpected. Notification will be made within 10 working days after the Coordinating Site becomes aware of the event.

Each principal investigator is responsible for reporting serious study-related adverse events and abiding by any other reporting requirements specific to his/her Institutional Review Board or Ethics Committee. Where the JCHR IRB is the overseeing IRB, sites must report all serious, related adverse events within seven (7) calendar days.

Upon receipt of a qualifying event, the Sponsor will investigate the event to determine if a UADE is confirmed, and if indicated, report the results of the investigation to all overseeing IRBs, and the FDA within 10 working days of the Sponsor becoming aware of the UADE per 21CFR 812.46(b) (2). The Medical Monitor must determine if the UADE presents an unreasonable risk to participants. If so, the Medical Monitor must ensure that all investigations, or parts of investigations presenting that risk, are terminated as soon as possible but no later than 5 working days after the Medical Monitor makes this determination and no later than 15 working days after first receipt notice of the UADE.

Device malfunctions will be handled by the Sponsor or designee as described below. In the case of a device malfunction, information will be forwarded to Insulet by the study staff to be addressed by approved company personnel.

6.5 Safety Oversight

The study Medical Monitor will review all adverse events and adverse device events that are reported during the study. Severe adverse events (SAE) typically will be reviewed within 24 hours of reporting. Other AEs typically will be reviewed on a weekly basis. Additionally, the Medical Monitor will review compiled safety data at the end of phase 1 (n=9) and phase 2 (n=9).

Safety parameter review: assessment for any of the below attributed to sensor error or inappropriate insulin delivery by the AID system will be reviewed in aggregate after each phase.

- Severe hypoglycemia is defined as severe if the event required assistance of another person to actively administer carbohydrate, glucagon, or other resuscitative actions due to altered consciousness.
- UADE: means any serious adverse effect on health or safety or any life-threatening problem or death caused by, or associated with, a device, if that effect, problem, or death was not previously identified in nature, severity, or degree of incidence in the investigational plan or application (including a supplementary plan or application), or any other unanticipated serious problem associated with a device that relates to the rights, safety, or welfare of subjects.
- Diabetic Ketoacidosis (requires all four criteria to be met):
    1. Symptoms such as polyuria, polydipsia, nausea, or vomiting
    2. Serum ketones > 1.5 mmol/L or large/moderate urine ketones
    3. Either arterial blood pH < 7.30 or venous pH < 7.24 or serum bicarbonate < 15
    4. DKA is suspected as either the primary or a contributing cause for these findings

The Clinical Study Director will be informed of all cases of severe hypoglycemia and DKA and the Medical Monitor's assessment of relationship to the study device; and informed of all reported device issues.

6.6 Stopping Criteria

6.6.1 Participant Discontinuation of Study Device

In the case of an unanticipated system malfunction resulting in a severe hypoglycemia or DKA event (or a malfunction that could have led to severe hypoglycemia or DKA), use of the HCL system will be suspended while the problem is diagnosed. The UADE will be reported to the IRB and the FDA. After assessment of the problem and any correction, use of the system will not be restarted until approval is received from the IRB and the FDA.

HCL therapy will be discontinued and patients will be transitioned to standard subcutaneous insulin therapy with continued management by the primary medical/surgical team or inpatient endocrine consult service (as clinically warranted) if any of the following occur:

- The investigator believes it is unsafe for the participant to continue on the intervention. This could be due to the development of a new medical condition or worsening of an existing condition; or participant behavior contrary to the indications for use of the device that imposes on the participant's safety
- Participant's clinical team feels transition to IV insulin infusion or MDI regimen is necessary for glycemic control
- A successful CGM validation has not been achieved per the protocol detailed in section *3.1.1* on two consecutive CGM sensors
- The participant requests that the treatment be stopped

- Participant pregnancy
- Participant requires hemodialysis or continuous renal replacement therapy (CRRT)
- Participant requires treatment with hydroxyurea or high dose ascorbic acid
- One episode of DKA as defined in section *6.2.4*
- One episode of severe hypoglycemia as defined in section *6.2.3*
- One episode of DKA as defined in section *6.2.4* and one severe hypoglycemia event as defined in section *6.2.3*
- Participant is anticipated to be discharged from the hospital without plan to continue HCL insulin therapy in outpatient setting; transition to subcutaneous insulin regimen will be initiated prior to discharge.

Transfers to the ICU will prompt discontinuation of use of the HCL component of the system (CGM may be used at described in section *3.2.1*). However, participant may be transitioned back to HCL therapy after transferring out of the ICU to a non-ICU ward based on study team assessment. ICU transfers will not be reported as AE or ADE unless the specific reason for transfer is glycemia-related (i.e. severe hypoglycemia or DKA).

6.6.2 Criteria for Suspending or Stopping Overall Study

In addition to the suspension of device use due to a UADE as described in section *6.6.1*, study activities could be similarly suspended if the manufacturer of any constituent study device requires stoppage of device use for safety reasons (e.g. product recall). The affected study activities may resume if the underlying problem can be corrected by a protocol or system modification that will not invalidate the results obtained prior to suspension.

Additionally, the entire study will be placed on hold pending further review and recommendations by the Medical Monitor and IRB for any of the following:

- Unanticipated adverse events attributable to study device malfunction (including DKA or seizure), and not resulting from known potential infusion site issues.
- Two patients with ≥1 episode of severe hypoglycemia attributable to insulin delivered by study device.

Chapter 7: Miscellaneous Considerations

7.1 Drugs Used as Part of the Protocol

Participants will be limited to the insulin approved for pump use (i.e. insulin lispro or insulin aspart).

7.2 Collection of Medical Conditions and Medications

Pre-Existing Condition: Any medical condition that is either present at screening, a chronic disease, or a prior condition that could impact the participant's health during the course of the study (e.g., prior myocardial infarction or stroke).

Medical Conditions during the study: In addition to conditions meeting the reporting requirements for an adverse event or device issue as described above, the following medical conditions should also be reported: (1) new diagnosis of a chronic disease (i.e., not present at the time of enrollment), and (2) any medical condition that could affect the participant's ability to carry out any aspect of the protocol or could affect an outcome assessment.

Medications: All medication for the treatment of chronic pre-existing conditions, medical conditions (including medical conditions that do not require recording), and/or adverse events that the participant is currently taking at screening and during the course of the study should be recorded. Nutraceuticals and preventative treatment also should be recorded. Medications only taken as needed will only be recorded if used during the study (e.g. Glucagon for treatment of severe hypoglycemia).

7.3 Prohibited Medications, Devices, Treatments, and Procedures

Non-insulin antihyperglycemics: non-insulin agents will be discontinued at enrollment

Additional insulins: Treatment of *hyperglycemia* with additional insulin beyond what is delivered by HCL therapy will not be permitted in conjunction with ongoing HCL use. For participants who require alternative or additional insulin therapy at the discretion of the patient's inpatient providers, the HCL system will be discontinued, as described in section *6.6.1*. Emergency use on insulins for *non-glucose-related indications*, such as hyperkalemia, are permitted; however, HCL therapy may be temporarily suspended at the discretion of the investigator and subsequently resumed as described in section *3.2.1*.

Hydroxyurea: Participants who require treatment with hydroxyurea will discontinue HCL therapy, as described in section *6.6.1*.

High-dose ascorbic acid (>1g/day): Participants who require treatment with ascorbic acid will discontinue HCL therapy, as described in section *6.6.1*.

All study devices (insulin pump, CGM) must be removed before Magnetic Resonance Imaging (MRI) or diathermy treatment. CGM equipment may be worn during Computed Tomography (CT).

7.4 Precautionary Medications, Treatments, and Procedures

Acetaminophen doses of >1 gram every 6 hours may cause CGM readings to be artificially elevated. Acetaminophen use should be limited to ≤4 grams per day.

7.5 Prophylactic Medications, Treatments, and Procedures

This section is not applicable to this study.

7.6 Rescue Medications, Treatments, and Procedures

All rescue therapies are allowed at the discretion of the participant's inpatient providers. HCL therapy should be discontinued in the event that certain medications are required, as described in sections *3.2.1*, *6.6.1*, and *7.3*.

7.7 Pregnancy Reporting

Pregnancy occurring during this study is highly unlikely. However, if pregnancy is discovered after enrollment, the participant will be discontinued from the study. The occurrence of pregnancy will be reported to the Coordinating Site and to the JCHR IRB as an Unanticipated Problem within seven (7) calendar days.

7.8 Participant Compensation

Participant compensation will be specified in the informed consent form.

7.9 Participant Withdrawal

Participation in the study is voluntary, and a participant may withdraw at any time. For participants who withdraw, their data will be used up until the time of withdrawal.

7.10 Confidentiality

For security and confidentiality purposes, participants will be assigned an identifier that will be used instead of their name. Protected health information gathered for this study will be shared with the Statistical Center, the Jaeb Center for Health Research in Tampa, FL. De-identified participant information may also be provided to research sites involved in the study.

Chapter 8: Statistical Considerations

8.1 Statistical and Analytical Plans

The approach to sample size and statistical analyses are summarized below.

8.2 Statistical Hypotheses

This is a pilot and feasibility trial and is not powered to reach statistical conclusions.

8.3 Sample Size

The sample size was a convenient sample size by which to obtain preliminary data on implementing the HCL/AID system in a hospital setting. The maximum number of participants to be consented for participation in this study (n=30) is based upon the goal of enrolling 18 patients with at least 48 hours of data during HCL therapy and to account for the potential for early device wear termination, ICU transfer, or early discharge.

8.4 Outcome Measures

Primary Endpoints:

Aim 1: Proportion of time spent in HCL after CGM validation

Aim 2: Percentage of time sensor glucose is within target glucose range [time-in-range (TIR)], defined as sensor glucose 70-180 mg/dL.

Secondary Endpoints:

Aim 1.1: System function

- Time from enrollment to start of HCL therapy
- Percent of time with CGM readings
- Percentage of validations that were successful
- Number of CGM readings within %15/15 of POC readings and within %20/20 of POC readings with the cut point at 70 mg/dL.

Aim 1.2 Patient perception of AID use in the hospital (qualitative analyses):

- Patients survey and open-ended feedback

Aim 2.1: Glycemic control

- Number of hypoglycemic (<70 mg/dL) and clinically-important hypoglycemic (<54 mg/dL) episodes per patient and per patient-day
- Percent time below range (TBR, <70mg/dL)
- Percent time in clinically-important hypoglycemia (<54 mg/dL)
- Percent time above range (TAR, >180 mg/dL)

- Percent time in severe hyperglycemia (>250 mg/dL)
- Glycemic variability (CV and SD)

Aim 2.2: HCL Settings

- Frequency of setting adjustments for clinically-important hypoglycemia (<54 mg/dL): Overall, to basal rate, to ICR, to ISF
- Frequency of setting adjustments for prolonged severe hyperglycemia (>250 mg/dL for >1 hour), Overall, to basal rate, to ICR, to ISF
- Insulin requirements: Total daily insulin (TDI), Total daily basal insulin, Total daily bolus insulin

8.5 Analysis Dataset

All relevant participant data will be included in the analysis of a single data set for this feasibility study.

8.6 Analysis of the Primary Efficacy Endpoint(s)

The primary purpose of this pilot study is to assess the feasibility HCL use in the inpatient setting; therefore, no formal power calculations were performed. The main objectives of this study are to improve the quality and efficiency of the main study. In addition, it is conducted in order increase the investigators' experience with the technology in a different setting. We will list values for each patient as well as provide summary statistics appropriate to the distribution. To determine the operability of AID in the hospital (Aim 1) we will calculate the proportion of time spent in HCL after CGM sensor values meet validation criteria [sensor glucose values are within ±20% of POC values for glucose levels ≥70 mg/dL or ±20 mg/dL for POC glucose values <70 mg/dL]. To evaluate glycemic control (Aim 2) we will determine the percentage of time sensor glucose values are within target glucose range [time-in-range (TIR); 70-180 mg/dL], during closed-loop insulin delivery.

8.7 Analysis of the Secondary Endpoints

The analysis of secondary endpoints will parallel that described above for the primary analysis with summary statistics given for each of the metrics listed in Section 8.4

8.8 Safety Analyses

Safety endpoints will be analyzed using summary statistics during treatment. AEs will be coded as described in section 6.2.8. Severity and relationship of AE to study agent will be determined as described in section 6.2. The event start date, stop date, severity, relationship, outcome, and duration will also be recorded. The Medical Monitor will conduct a review of safety parameters at the end of each study phase, as described in Section 6.5.

The following will be tabulated during the feasibility study:

- Number of adverse events
- Number of participants with at least one event
- Number of serious adverse events
- Number of participants with at least one serious adverse event
- Number of unexpected device events
- Number of unexpected serious device events
- Number of ICU transfers
- Number of adverse events thought by investigator to be related to study device
- Number of participants who stopped the intervention in response to an adverse event
- Number of severe hypoglycemic events as defined in the protocol
- Number of severe hypoglycemic events associated with seizure or loss of consciousness
- Number of diabetic ketoacidosis events as defined in the protocol

8.9 Intervention Adherence

The adherence to the protocol will be determined by daily use of HCL after enrollment and will be calculated from time of enrollment to ICU transfer or hospital discharge. We will obtain preliminary estimates about the frequency and reasons for discontinuation of the intervention during this feasibility study.

8.10 Protocol Adherence and Retention

Potential study subjects will be identified and pre-screened among non-ICU patients with hyperglycemia requiring insulin therapy. Strategies to address potential problems with recruitment will include bi-weekly communications with Drs. Pasquel, Davis, Buckingham, Lal, and Brown.

A study coordinator or investigator will conduct informed consent in eligible subjects prior to any study procedures. Screening and recruitment reports will be generated monthly that include actual and expected recruitment statistics. Based on preliminary studies with inpatient use of CGM we expect to retain most patients in this study.

8.11 Baseline Descriptive Statistics

Baseline characteristics will be collected and reported for all participants in this feasibility study.

8.12 Device Issues

Any technical issues will be reported to Insulet Corporation. Any device issues affecting patient care will be reported as below and reviewed by the Medical Monitor.

8.13 Planned Interim Analyses

The criteria below will be considered events prompting an interim review and safety analysis prior to continuation of the study. The study investigators and statisticians will perform the

statistical analysis that will be reviewed by the Medical Monitor. There are no blinding procedures to be addressed in this feasibility study.

- Adverse events attributable to study device (including DKA or seizure)
- Two patients with ≥1 episode of severe hypoglycemia attributable to insulin delivered by study device.

8.14 Sub-Group Analyses

Not applicable in this feasibility study.

8.15 Multiple Comparison/Multiplicity

Not applicable in this single-arm feasibility study.

8.16 Exploratory Analyses

No additional exploratory analyses are planned.

8.17 Additional Tabulations and Analyses

Not applicable in this single-arm feasibility study.

Chapter 9: Data Collection and Monitoring

9.1 Case Report Forms and Other Data Collection

The main study data are collected on electronic case report forms (eCRFs). When data are directly collected in electronic case report forms, this will be considered the source data. For any data points for which the eCRF is not considered source (e.g. lab results that are transcribed from a printed report into the eCRF), the original source documentation must be maintained in the participant's study chart or medical record. This source must be readily verifiable against the values entered into eCRF. Even where all study data are directly entered into the eCRFs at office visits, evidence of interaction with a live subject must be recorded (e.g., office note, visit record, etc.)

Electronic device data files are obtained from the study software and individual hardware components. These electronic device files are considered the primary source documentation.

9.2 Study Records Retention

Each participating site will maintain appropriate medical and research records for this trial, in compliance with ICH E6 and regulatory and institutional requirements for the protection of confidentiality of participants.

Study documents should be retained for a minimum of 3 years after final reporting. These documents should be retained for a longer period, however, if required by local regulations. No records will be destroyed without the written consent of the sponsor, if applicable. It is the responsibility of the sponsor to inform the investigator when these documents no longer need to be retained.

9.3 Quality Assurance and Monitoring

Designated personnel from Emory University will be responsible for maintaining quality assurance (QA) and quality control (QC) systems to ensure that the clinical portion of the trial is conducted and data are generated, documented and reported in compliance with the protocol, Good Clinical Practice (GCP) and the applicable regulatory requirements, as well as to ensure that the rights and wellbeing of trial participants are protected and that the reported trial data are accurate, complete, and verifiable. Adverse events will be prioritized for monitoring.

A risk-based monitoring (RBM) plan will be developed and revised as needed during the course of the study, consistent with the FDA "Guidance for Industry Oversight of Clinical Investigations — A Risk-Based Approach to Monitoring" (August 2013). Study conduct and monitoring will conform with 21 Code of Federal Regulations (CFR) 812. This plan describes in detail who will conduct the monitoring, at what frequency monitoring will be done, at what level of detail monitoring will be performed, and the distribution of monitoring reports.

The data of most importance for monitoring at the site are participant eligibility and adverse events. Therefore, the RBM plan will focus on these areas. As much as possible, remote

monitoring will be performed in real-time with on-site monitoring performed to evaluate the verity and completeness of the key site data. Elements of the RBM may include:

- Qualification assessment, training, and certification for sites and site personnel
- Oversight of Institutional Review Board (IRB) coverage and informed consent procedures
- Central (remote) data monitoring: validation of data entry, data edits/audit trail, protocol review of entered data and edits, statistical monitoring, study closeout
- On-site monitoring (site visits): source data verification, site visit report
- Agent/Device accountability
- Communications with site staff
- Patient retention and visit completion
- Quality control reports
- Management of noncompliance
- Documenting monitoring activities
- Adverse event reporting and monitoring

Coordinating Site (Emory University) representatives or their designees may visit the study facilities at any time in order to maintain current and personal knowledge of the study through review of the records, comparison with source documents, observation and discussion of the conduct and progress of the study. The investigational site will provide direct access to all trial related sites, source data/documents, and reports for the purpose of monitoring and auditing by the sponsor, and inspection by local and regulatory authorities.

9.4 Protocol Deviations

A protocol deviation is any noncompliance with the clinical trial protocol, GCP, or procedure requirements. The noncompliance may be either on the part of the participant, the investigator, or the study site staff. As a result of deviations, corrective actions are to be developed by the site and implemented promptly.

The site PI/study staff is responsible for knowing and adhering to their IRB requirements. Further details about the handling of protocol deviations will be included in the monitoring plan.

Chapter 10: Ethics/Protection of Human Participants

10.1 Ethical Standard

The investigator will ensure that this study is conducted in full conformity with Regulations for the Protection of Human Participants of Research codified in 45 CFR Part 46, 21 CFR Part 50, 21 CFR Part 56, and/or the ICH E6.

10.2 Institutional Review Boards

The protocol, informed consent form(s), recruitment materials, and all participant materials will be submitted to the JCHR IRB for review and approval as the IRB of record. Approval of both the protocol and the consent form must be obtained before any participant is enrolled. Any amendment to the protocol will require review and approval by the JCHR IRB before the changes are implemented to the study. All changes to the consent form will be JCHR IRB approved; a determination will be made regarding whether previously consented participants need to be re-consented.

10.3 Informed Consent Process

10.3.1 Consent Procedures and Documentation

Informed consent is a process that is initiated prior to the individual's agreeing to participate in the study and continues throughout the individual's study participation. A partial HIPAA waiver will be obtained to review hospital admissions for potential participants in this trial. Those meeting inclusion criteria will be approached by the study team and invited to participate. Extensive discussion of risks and possible benefits of participation will be provided to the participants and their families. Consent forms will be IRB-approved and the participant will be asked to read and review the document. The investigator will explain the research study to the participant and answer any questions that may arise. All participants will receive a verbal explanation in terms suited to their comprehension of the purposes, procedures, and potential risks of the study and of their rights as research participants. Participants will have the opportunity to carefully review the written consent form and ask questions prior to signing.

The participants should have the opportunity to discuss the study with their surrogates or think about it prior to agreeing to participate. The participant will sign the informed consent document prior to any procedures being done specifically for the study. The participants may withdraw consent at any time throughout the course of the trial. A copy of the informed consent document will be given to the participants for their records. The rights and welfare of the participants will be protected by emphasizing to them that the quality of their medical care will not be adversely affected if they decline to participate in this study.

10.3.2 Participant and Data Confidentiality

Participant confidentiality is strictly held in trust by the participating investigators, their staff, and the sponsor(s) and their agents. This confidentiality is extended to cover testing of biological samples and genetic tests in addition to the clinical information relating to participants. Therefore, the study protocol, documentation, data, and all other information generated will be

held in strict confidence. No information concerning the study or the data will be released to any unauthorized third party without prior written approval of the sponsor.

The study monitor, other authorized representatives of the sponsor, representatives of the IRB, regulatory agencies or company supplying study product may inspect all documents and records required to be maintained by the investigator, including but not limited to, medical records (office, clinic, or hospital) and pharmacy records for the participants in this study. The clinical study site will permit access to such records.

The study participant's contact information will be securely stored at each clinical site for internal use during the study. At the end of the study, all records will continue to be kept in a secure location for as long a period as dictated by the reviewing IRB, institutional policies, or sponsor requirements.

Study participant research data, which is for purposes of statistical analysis and scientific reporting, will be transmitted to and stored at the Coordinating Site and Statistical Center. This will not include the participant's contact or identifying information, unless otherwise specified in the informed consent form. Rather, individual participants and their research data will be identified by a unique study identification number. The study data entry and study management systems used by clinical sites will be secured and password protected. At the end of the study, all study databases will be de-identified and archived.

10.3.3 Future Use of Stored Specimens and Data

Biologic samples from participants will not be stored for the purposes of this study. Genetic testing will not be performed as part of this study.

Chapter 11: References

1. Pasquel FJ, Umpierrez GE. Individualizing Inpatient Diabetes Management During the Coronavirus Disease 2019 Pandemic. *J Diabetes Sci Technol.* 2020;14(4):705-707.
2. Team CC-R. Preliminary Estimates of the Prevalence of Selected Underlying Health Conditions Among Patients with Coronavirus Disease 2019 - United States, February 12-March 28, 2020. *MMWR Morb Mortal Wkly Rep.* 2020;69(13):382-386.
3. Killerby ME, Link-Gelles R, Haight SC, et al. Characteristics Associated with Hospitalization Among Patients with COVID-19 - Metropolitan Atlanta, Georgia, March-April 2020. *MMWR Morb Mortal Wkly Rep.* 2020;69(25):790-794.
4. Gold JAW, Wong KK, Szablewski CM, et al. Characteristics and Clinical Outcomes of Adult Patients Hospitalized with COVID-19 - Georgia, March 2020. *MMWR Morb Mortal Wkly Rep.* 2020;69(18):545-550.
5. Auld S, Caridi-Scheible M, Blum JM, et al. ICU and ventilator mortality among critically ill adults with COVID-19. *medRxiv.* 2020.
6. Bally L, Thabit H, Hartnell S, et al. Closed-Loop Insulin Delivery for Glycemic Control in Noncritical Care. *N Engl J Med.* 2018;379(6):547-556.
7. Boughton CK, Bally L, Martignoni F, et al. Fully closed-loop insulin delivery in inpatients receiving nutritional support: a two-centre, open-label, randomised controlled trial. *Lancet Diabetes Endocrinol.* 2019;7(5):368-377.
8. Thabit H, Hartnell S, Allen JM, et al. Closed-loop insulin delivery in inpatients with type 2 diabetes: a randomised, parallel-group trial. *Lancet Diabetes Endocrinol.* 2017;5(2):117-124.
9. Vanhorebeek I, Van den Berghe G. Hormonal and metabolic strategies to attenuate catabolism in critically ill patients. *Curr Opin Pharmacol.* 2004;4(6):621-628.
10. Van den Berghe G. How does blood glucose control with insulin save lives in intensive care? *J Clin Invest.* 2004;114(9):1187-1195.
11. van den Berghe G, Wouters P, Weekers F, et al. Intensive insulin therapy in the critically ill patients. *N Engl J Med.* 2001;345(19):1359-1367.
12. Van den Berghe G, Wouters PJ, Bouillon R, et al. Outcome benefit of intensive insulin therapy in the critically ill: Insulin dose versus glycemic control. *Crit Care Med.* 2003;31(2):359-366.
13. Finfer S, Chittock DR, Su SY, et al. Intensive versus conventional glucose control in critically ill patients. *N Engl J Med.* 2009;360(13):1283-1297.
14. Investigators N-SS, Finfer S, Liu B, et al. Hypoglycemia and risk of death in critically ill patients. *N Engl J Med.* 2012;367(12):1108-1118.
15. Wintergerst KA, Buckingham B, Gandrud L, Wong BJ, Kache S, Wilson DM. Association of hypoglycemia, hyperglycemia, and glucose variability with morbidity and death in the pediatric intensive care unit. *Pediatrics.* 2006;118(1):173-179.
16. Kulkarni H, Bihari S, Prakash S, et al. Independent Association of Glucose Variability With Hospital Mortality in Adult Intensive Care Patients: Results From the Australia and New Zealand Intensive Care Society Centre for Outcome and Resource Evaluation Binational Registry. *Crit Care Explor.* 2019;1(8):e0025.
17. Chao WC, Tseng CH, Wu CL, Shih SJ, Yi CY, Chan MC. Higher glycemic variability within the first day of ICU admission is associated with increased 30-day mortality in ICU patients with sepsis. *Ann Intensive Care.* 2020;10(1):17.

18. Zhu L, She ZG, Cheng X, et al. Association of Blood Glucose Control and Outcomes in Patients with COVID-19 and Pre-existing Type 2 Diabetes. *Cell Metab.* 2020;31(6):1068-1077 e1063.
19. Sardu C, D'Onofrio N, Balestrieri ML, et al. Outcomes in Patients With Hyperglycemia Affected by COVID-19: Can We Do More on Glycemic Control? *Diabetes Care.* 2020;43(7):1408-1415.
20. Pasquel FJ, Fayfman M, Umpierrez GE. Debate on Insulin vs Non-insulin Use in the Hospital Setting-Is It Time to Revise the Guidelines for the Management of Inpatient Diabetes? *Curr Diab Rep.* 2019;19(9):65.
21. Rayman G, Lumb AN, Kennon B, et al. Dexamethasone therapy in COVID-19 patients: implications and guidance for the management of blood glucose in people with and without diabetes. *Diabetic Medicine.* 2020;n/a(n/a).
22. Horby P, Lim WS, Emberson JR, et al. Dexamethasone in Hospitalized Patients with Covid-19 - Preliminary Report. *N Engl J Med.* 2020.
23. Davis GM, Galindo RJ, Migdal AL, Umpierrez GE. Diabetes Technology in the Inpatient Setting for Management of Hyperglycemia. *Endocrinol Metab Clin North Am.* 2020;49(1):79-93.
24. Umpierrez GE, Klonoff DC. Diabetes Technology Update: Use of Insulin Pumps and Continuous Glucose Monitoring in the Hospital. *Diabetes Care.* 2018;41(8):1579-1589.
25. Galindo RJ, Aleppo G, Klonoff DC, et al. Implementation of Continuous Glucose Monitoring in the Hospital: Emergent Considerations for Remote Glucose Monitoring During the COVID-19 Pandemic. *J Diabetes Sci Technol.* 2020;14(4):822-832.
26. Migdal AL, Spanakis EK, Galindo RJ, et al. Accuracy and Precision of Continuous Glucose Monitoring in Hospitalized Patients Undergoing Radiology Procedures. *J Diabetes Sci Technol.* 2020:1932296820930038.
27. Pasquel FJ, Lansang MC, Khowaja A, et al. A Randomized Controlled Trial Comparing Glargine U300 and Glargine U100 for the Inpatient Management of Medicine and Surgery Patients With Type 2 Diabetes: Glargine U300 Hospital Trial. *Diabetes Care.* 2020;43(6):1242-1248.
28. Galindo RJ, Migdal AL, Davis GM, et al. Comparison of the FreeStyle Libre Pro Flash Continuous Glucose Monitoring (CGM) System and Point-of-Care Capillary Glucose Testing (POC) in Hospitalized Patients With Type 2 Diabetes (T2D) Treated With Basal-Bolus Insulin Regimen. *Diabetes Care.* 2020.
29. Teles M, Sacchetta T, Matsumoto Y. COVID-19 Pandemic Triggers Telemedicine Regulation and Intensifies Diabetes Management Technology Adoption in Brazil. *J Diabetes Sci Technol.* 2020;14(4):797-798.
30. Shehav-Zaltzman G, Segal G, Konvalina N, Tirosh A. Remote Glucose Monitoring of Hospitalized, Quarantined Patients With Diabetes and COVID-19. *Diabetes Care.* 2020;43(7):e75-e76.
31. Reutrakul S, Genco M, Salinas H, et al. Feasibility of Inpatient Continuous Glucose Monitoring During the COVID-19 Pandemic: Early Experience. *Diabetes Care.* 2020.
32. Singh LG, Satyarengga M, Marcano I, et al. Reducing Inpatient Hypoglycemia in the General Wards Using Real-Time Continuous Glucose Monitoring: The Glucose Telemetry System, a Randomized Clinical Trial. *Diabetes Care.* 2020.

33. Fortmann AL, Spierling Bagsic SR, Talavera L, et al. Glucose as the Fifth Vital Sign: A Randomized Controlled Trial of Continuous Glucose Monitoring in a Non-ICU Hospital Setting. *Diabetes Care.* 2020.
34. Lal RA, Basina M, Maahs DM, Hood K, Buckingham B, Wilson DM. One Year Clinical Experience of the First Commercial Hybrid Closed-Loop System. *Diabetes Care.* 2019;42(12):2190-2196.
35. Sherr JL, Buckingham BA, Forlenza GP, et al. Safety and Performance of the Omnipod Hybrid Closed-Loop System in Adults, Adolescents, and Children with Type 1 Diabetes Over 5 Days Under Free-Living Conditions. *Diabetes Technol Ther.* 2020;22(3):174-184.
36. Anderson SM, Buckingham BA, Breton MD, et al. Hybrid Closed-Loop Control Is Safe and Effective for People with Type 1 Diabetes Who Are at Moderate to High Risk for Hypoglycemia. *Diabetes Technol Ther.* 2019;21(6):356-363.
37. Lawton J, Blackburn M, Rankin D, et al. Participants' Experiences of, and Views About, Daytime Use of a Day-and-Night Hybrid Closed-Loop System in Real Life Settings: Longitudinal Qualitative Study. *Diabetes Technol Ther.* 2019;21(3):119-127.
38. Buckingham B, Brown SA, Bode B, Levy C, GP F, Criego A. Omnipod® 5 Automated Glucose Control System, Powered by Horizon™. American Diabetes Association Annual Scientific Meeting; June 12th, 2020, 2020; Chicago, Virtual.
39. Partridge H, Perkins B, Mathieu S, Nicholls A, Adeniji K. Clinical recommendations in the management of the patient with type 1 diabetes on insulin pump therapy in the perioperative period: a primer for the anaesthetist. *Br J Anaesth.* 2016;116(1):18-26.
40. Sobel SI, Augustine M, Donihi AC, Reider J, Forte P, Korytkowski M. Safety and efficacy of a peri-operative protocol for patients with diabetes treated with continuous subcutaneous insulin infusion who are admitted for same-day surgery. *Endocr Pract.* 2015;21(11):1269-1276.
41. Vanderhoek SM, Wolf RM. Use of continuous subcutaneous insulin infusion (CSII) therapy in pediatric diabetes patients in the perioperative period. *Paediatr Anaesth.* 2019;29(9):901-906.
